# Solvent-mediated precipitating synthesis and optical properties of polyhydrido Cu_13_ nanoclusters with four vertex-sharing tetrahedrons†

**DOI:** 10.1039/d2sc06099j

**Published:** 2022-12-20

**Authors:** Xinzhang Lin, Jie Tang, Chenyu Zhu, Li Wang, Yang Yang, Ren'an Wu, Hongjun Fan, Chao Liu, Jiahui Huang

**Affiliations:** a Dalian National Laboratory for Clean Energy, Dalian Institute of Chemical Physics, Chinese Academy of Sciences Dalian 116023 China chaoliu@dicp.ac.cn jiahuihuang@dicp.ac.cn; b Laboratory of High-Resolution Mass Spectrometry Technologies, Dalian Institute of Chemical Physics, Chinese Academy of Sciences Dalian 116023 China; c State Key Laboratory of Molecular Reaction Dynamics, Dalian Institute of Chemical Physics, Chinese Academy of Sciences Dalian 116023 China fanhj@dicp.ac.cn; d University of Chinese Academy of Sciences Beijing 100049 China

## Abstract

Structurally defined metal nanoclusters facilitate mechanism studies and promote functional applications. However, precisely constructing copper nanoclusters remains a long-standing challenge in nanoscience. Developing new efficient synthetic strategies for Cu nanoclusters is highly desirable. Here, we propose a solvent-mediated precipitating synthesis (SMPS) to prepare Cu_13_H_10_(SR)_3_(PPh_3_)_7_ nanoclusters (H–SR = 2-chloro-4-fluorobenzenethiol). The obtained Cu_13_ nanoclusters are high purity and high yield (39.5%, based on Cu atom), proving the superiority of the SMPS method. The Cu_13_ nanoclusters were comprehensively studied *via* a series of characterizations. Single crystal X-ray crystallography shows that the Cu_13_ nanoclusters contain a threefold symmetry axis and the Cu_13_ kernel is protected by a monolayer of ligands, including PPh_3_ and thiolates. Unprecedentedly, the aesthetic Cu_13_ kernel is composed of four vertex-sharing tetrahedrons, rather than the common icosahedral or cuboctahedral M_13_. The intramolecular π⋯π interactions between thiolates and PPh_3_ on the surface contribute to the stable configuration. Furthermore, electrospray ionization mass spectrometry (ESI-MS) and nuclear magnetic resonance (NMR) revealed the existence of ten hydrides, including four types of hydrides. Density functional theory (DFT) calculations without simplifying the ligands simulated the location of the 10 hydrides in the crystal structure. Additionally, the steady-state ultraviolet-visible absorption and fluorescence spectra of the Cu_13_ nanoclusters exhibit unique optical absorbance and photoluminescence. The ultrafast relaxation dynamics were also studied *via* transient absorption spectroscopy, and the three decay components are attributed to the relaxation pathways of internal conversion, structural relaxation and radiative relaxation. This work provides not only a novel SMPS strategy to efficiently synthesize Cu_13_ nanoclusters, but also a better insight into the structural characteristics and optical properties of the Cu nanoclusters.

## Abstract 2

A solvent-mediated precipitating synthesis was developed to efficiently prepare novel high-purity Cu_13_H_10_(SR)_3_(PPh_3_)_7_ nanoclusters with an aesthetic crystal structure, unique optical properties and ultrafast relaxation dynamics.

## Graphical abstract

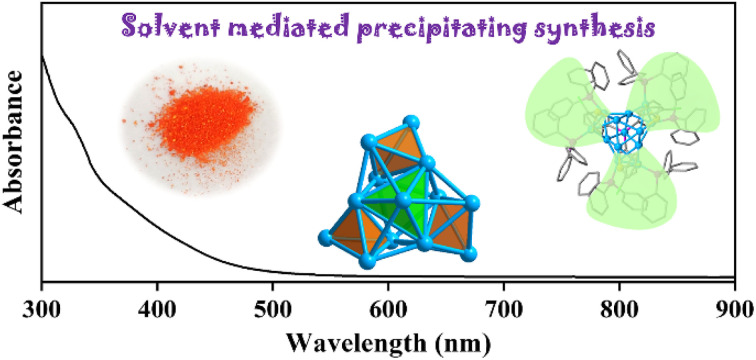

## Introduction

Copper has exerted a profound influence on human society since the Bronze Age. Atomically precise copper nanoclusters protected by organic ligands have been on the upsurge in nanoscience during the last decade.^1–5^ Copper-based nanomaterials play a crucial role in the applications of energy conversion,^6,7^ bio-imaging^8^ and chemical sensing,^9^*etc.* For example, copper is the dominant metal catalyst for the production of hydrocarbons through the electrochemical reduction of CO_2_.^10,11^ Additionally, metal nanoclusters exhibit a discrete electronic structure originating from quantum size effects and provide a novel opportunity for engineering their abundant molecular-like properties.^12–14^ The well-defined atomic composition and precise crystal structure of nanoclusters are beneficial for exploring their formation mechanism and catalytic mechanism and constructing structure–property relationships at the atomic level.^15,16^ Therefore, the controllable synthesis and structure determination of copper nanoclusters are of critical importance in nanotechnology. Over the decades, tremendous progress has been achieved in organic-ligand-protected Au and Ag nanoclusters, such as Au_25_,^17–19^ Au_40_,^20–22^ Au_60_,^23,24^ Au_92_,^25,26^ Au_110_,^27^ Au_279_,^28^ Ag_25_,^29^ Ag_78_,^14,30^ Ag_141_,^31^ Ag_374_,^32^*etc.* In contrast, the number of Cu nanoclusters is extremely limited owing to the high susceptibility towards oxidation of Cu. A handful of ligand-protected Cu nanoclusters have been synthesized and structurally characterized, such as Cu_11_,^33,34^ Cu_13_,^35–38^ Cu_15_,^4^ Cu_18_,^39,40^ Cu_20_,^41–43^ Cu_23_,^5,44,45^ Cu_25_,^40,46,47^ Cu_28_,^48^ Cu_32_,^49,50^ Cu_36_,^1^ Cu_53_,^51,52^ Cu_61_,^53^ Cu_81_,^54^*etc.* Nevertheless, compared with Au and Ag nanoclusters, the library of Cu nanoclusters requires further enrichment and expansion. However, the efficient synthesis of Cu nanoclusters still remains challenging.

The synthetic methods for Cu nanoclusters have generally followed those of Au and Ag nanoclusters. The Brust–Schiffrin method,^55^ a general strategy for Au nanoclusters, is also appropriate to synthesize Cu nanoclusters. Chen *et al.* prepared mixed-size Cu_*n*_ (*n* ≤ 8) nanoclusters using this method.^56^ Moreover, Sun *et al.* utilized the gradient reduction strategy with Cu powder and Ph_2_SiH_2_ as reductants, respectively, to obtain Cu_23_ nanoclusters.^5^ Zheng *et al.* designed a diamine-assisted synthetic strategy to construct Cu_32_ nanoclusters in an inert atmosphere.^50^ Bakr *et al.* presented a one-pot direct reduction strategy to synthesize Cu_81_ nanoclusters.^54^ Additionally, some other synthetic approaches for Cu nanoclusters have also been reported, including a water-in-oil microemulsion strategy, electrochemical synthesis, microwave-assisted polyol synthesis, *etc.*^57,58^ Although researchers have tried their best to develop the synthesis of Cu nanoclusters, effective synthetic strategies to obtain more Cu nanoclusters are still lacking. In addition, difficult separation, low yields and complicated procedures are limitations in the synthetic process. Therefore, designing and developing novel synthetic strategies to efficiently boost the purity and yield of Cu nanoclusters is of great importance and significance.

Herein, we develop a solvent-mediated precipitating synthesis (SMPS) to prepare a polyhydrido Cu nanocluster formulated as Cu_13_H_10_(SR)_3_(PPh_3_)_7_ (Cu_13_ for short). The SMPS is an efficient strategy to synthesize high-purity Cu_13_ nanoclusters in high yield. X-ray crystallographic analysis revealed that Cu_13_ nanoclusters have a threefold symmetry axis and comprise a Cu_13_ kernel with three types of exterior ligands. The Cu_13_ kernel consists of four vertex-sharing tetrahedrons, making it quite different from the common icosahedral or cuboctahedral M_13_ kernels. The intramolecular π⋯π interactions between thiolates and PPh_3_ are favorable to the configurational stability. ESI-MS and NMR analysis confirmed the molecular composition of the Cu_13_ nanoclusters including the number and type of hydrides. DFT was carried out to simulate the UV-vis absorption, electronic structure and location of the 10 hydrides for the nanocluster. Of note, to obtain accurate results, DFT calculations were performed using the entire structure of the Cu_13_ nanoclusters, without the conventional substitution of H–SCH_3_ for H–SR. Furthermore, the UV-vis absorption spectrum of the Cu_13_ nanoclusters exhibits multiple broad absorption peaks, while the emission spectrum exhibits three obvious peaks at 408, 434 and 458 nm, respectively. The transient absorption spectra revealed the ultrafast excited-state dynamics of the Cu_13_ nanoclusters with three relaxation pathways.

## Experimental section

### Chemicals and materials

All the following reagents were purchased and used as received without further purification. Cuprous bromide (CuBr, 99%), sodium borohydride (NaBH_4_, 98%), methanol (CH_3_OH, analytical grade), toluene (C_7_H_8_, analytical grade) and *n*-hexane (C_6_H_14_, chromatographic grade) were purchased from Sinopharm Chemical Reagent Co. Ltd. Acetonitrile (CH_3_CN, analytical grade) and dichloromethane (CH_2_Cl_2_, analytical grade) were purchased from Kermel. 2-Chloro-4-fluorobenzenethiol (C_6_H_4_ClFS, 97%) and triphenylphosphine (PPh_3_, 99%) were purchased from Alfa Aesar. All glassware was cleaned with *aqua regia* (v(HCl)/v(HNO_3_) = 3 : 1), rinsed with ultrapure water, and then dried in an oven prior to use.

### Synthesis of Cu_13_H_10_(SR)_3_(PPh_3_)_7_ nanoclusters

The Cu_13_ nanoclusters were prepared *via* a one-pot synthesis. 20 mg CuBr was dissolved in 10 mL CH_3_CN in a round-bottom flask. The solution was stirred vigorously using a magnetic stirrer at room temperature. After about five minutes, 5 mL CH_3_OH and 5 mL CH_2_Cl_2_ were added to the above solution. Subsequently, 37 mg PPh_3_ was added. Then, 17 μL of 2-chloro-4-fluorobenzenethiol was added. After about 20 minutes, 100 mg NaBH_4_ was added directly to the solution. About 10 minutes later, an orange-red precipitate was observed in the solution. After about 1 h, the precipitate was the crude product, and impurities in the supernatant were removed after centrifugation. Finally, the precipitate was dissolved in CH_2_Cl_2_ to extract the pure product. Of note, the product can also be prepared using CuCl or CuCl_2_·2H_2_O to replace CuBr, but the yield is lower than using CuBr.

### Synthesis of Cu_13_D_10_(SR)_3_(PPh_3_)_7_ nanoclusters

The deuterated Cu_13_ nanoclusters were prepared using the same procedure as the Cu_13_H_10_(SR)_3_(PPh_3_)_7_ nanoclusters *via* replacing NaBH_4_ with NaBD_4_.

### Single-crystal X-ray crystallography

Red block crystals of the Cu_13_ nanoclusters were grown in a mixture of CH_2_Cl_2_ and hexane in a 4 °C refrigerator. The X-ray crystallographic data of the Cu_13_ nanoclusters was collected using a single crystal X-ray diffractometer (Bruker D8 VENTURE) using Cu Kα radiation (*λ* = 1.54178 Å) at Shanghai Jiao Tong University. The structure was solved by direct methods and refined with full-matrix least squares on F2 using the SHELXTL software package and Olex2. All non-hydrogen atoms were refined anisotropically.^59–61^ CCDC deposition number: 2170868.

### Other physical measurements

The UV-vis spectra of Cu_13_ nanoclusters dissolved in CH_2_Cl_2_ were collected using a SHIMADZU UV-1800 spectrophotometer. The fluorescence spectra of the Cu_13_ nanoclusters were obtained using an Agilent Technologies Cary Eclipse Fluorescence Spectrophotometer. The electrospray ionization mass spectrum (ESI-MS) of the nanoclusters dissolved in a mixture of toluene and methanol (v/v = 1/1) was collected using a Fourier Transform Ion Cyclotron Resonance Mass Spectrometer (SolariX XR-15T). Digital photos of the solution and crystals of the Cu_13_ nanoclusters were shot using an MI 8 AI dual camera. XPS was conducted using a Thermo Fisher ESCALAB 250xi. ^1^H NMR and ^2^H NMR spectroscopy were carried out using a Bruker AVANCE 400 MHz and AVANCE III HD 700 MHz, respectively.

### Computational method

Density function theory calculations (DFT) were performed with the Gaussian 16 program package.^62^ The structural optimizations were carried out with the wb97xd functional^63^ and def2-SVP basis set.^64^ NMR shielding tensors were computed using the Gauge-Independent Atomic Orbital (GIAO) method on the optimized geometries, and the chemical shifts were calculated using tetramethylsilane as the reference.^65^ The electronic excitations were calculated using the time-dependent DFT method on the optimized geometries, and the UV-vis spectrum was simulated using a Gaussian broadening function with a full width at half maximum of 0.66667 eV.^66^

## Results and discussion

### Synthesis and crystallization

The Cu_13_H_10_(SR)_3_(PPh_3_)_7_ nanoclusters were obtained *via* a solvent-mediated precipitating synthesis. First, CuBr was dissolved in CH_3_CN, followed by the addition of CH_3_OH and CH_2_Cl_2_ in the proper order. Next, PPh_3_ and 2-chloro-4-fluorobenzenethiol were added successively. Finally, NaBH_4_ was added directly to the above solution to reduce the Cu–ligand complexes. About 10 minutes later, an orange-red precipitate appeared in the solution (Fig. S1a†). After centrifugation, the supernate containing the excess or unreacted reagents was removed, and the precipitate was the target product. The yield of the product was high (39.5% based on Cu atom). It is worth mentioning that the prepared product was of high purity without further washing, proving the superiority of the SMPS strategy. Choosing different types of solvent is beneficial to the interfacial reaction and precipitation of the Cu nanoclusters. This method might represent a strategy to synthesize less-stable metal nanoclusters *via* modulating the reaction solvents, in which the metal nanoclusters have different solubilities. Red block crystals of the Cu_13_ nanoclusters (Fig. S1b–d†) were grown in a mixture of CH_2_Cl_2_ and hexane in a 4 °C refrigerator for 1–2 days (for details, see Experimental section). Using NaBD_4_ to replace NaBH_4_, the deuterated analogue (Cu_13_D_10_(SR)_3_(PPh_3_)_7_, Cu_13D_ for short) was synthesized following the same procedure.

### Crystal structure

**Fig. 1:**
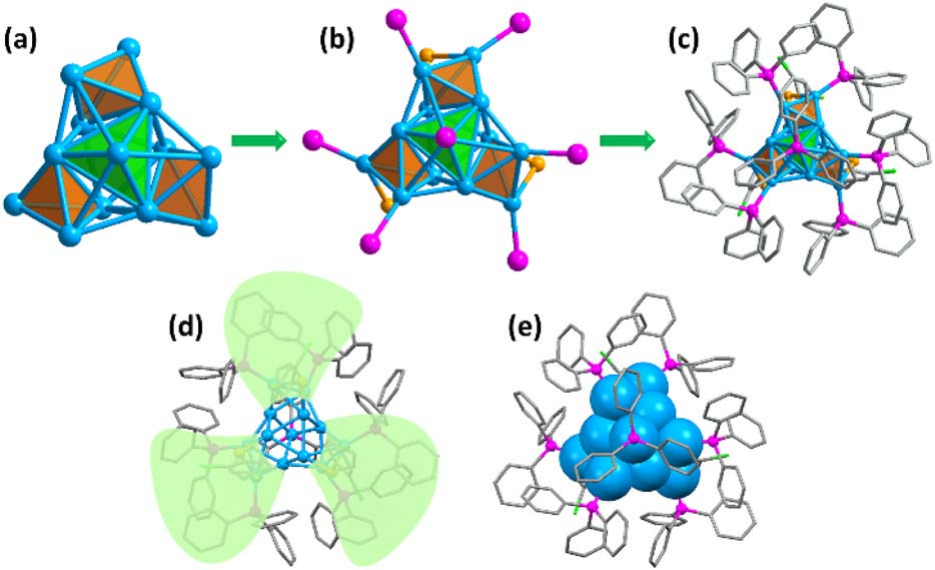
(a) The Cu_13_ kernel composed of four vertex-sharing tetrahedrons. (b) The Cu_13_ kernel with P and S atoms. (c–e) The total crystal structure of the Cu_13_H_10_(SR)_3_(PPh_3_)_7_ nanoclusters presented in different styles. The three tetrahedrons at the bottom are marked in orange and the tetrahedron at the top is marked in green. Color code: blue, Cu; magenta, P; orange, S; green, Cl; grey, C. The hydrogen atoms are omitted for clarity.

The crystal structure of the Cu_13_ nanoclusters was determined using single crystal X-ray crystallography. The Cu_13_ nanoclusters crystallized in the trigonal crystal system with the *R*3̄ space group, and twelve Cu_13_ nanoclusters are packed in a unit cell (Table S1 and Fig. S2†). Fig. 1 shows the total crystal structure of the Cu_13_ nanoclusters with a threefold symmetry (C_3_) axis. Four tetrahedrons constitute the aesthetic Cu_13_ kernel *via* sharing three vertices, which is different from the common M_13_ icosahedron or cuboctahedron structures (Fig. 1a). The tetrahedron at the top marked in green occupies the C_3_ axis, and the three tetrahedrons in the bottom marked in orange are distributed symmetrically along the C_3_ axis. The Cu_13_ metal kernel is encapsulated by 7 PPh_3_ and 3 thiolates, and the total structure looks like a tri-blade fan (Fig. 1b–e), which is similar to that of Cu_15_ nanoclusters.^4^

**Fig. 2:**
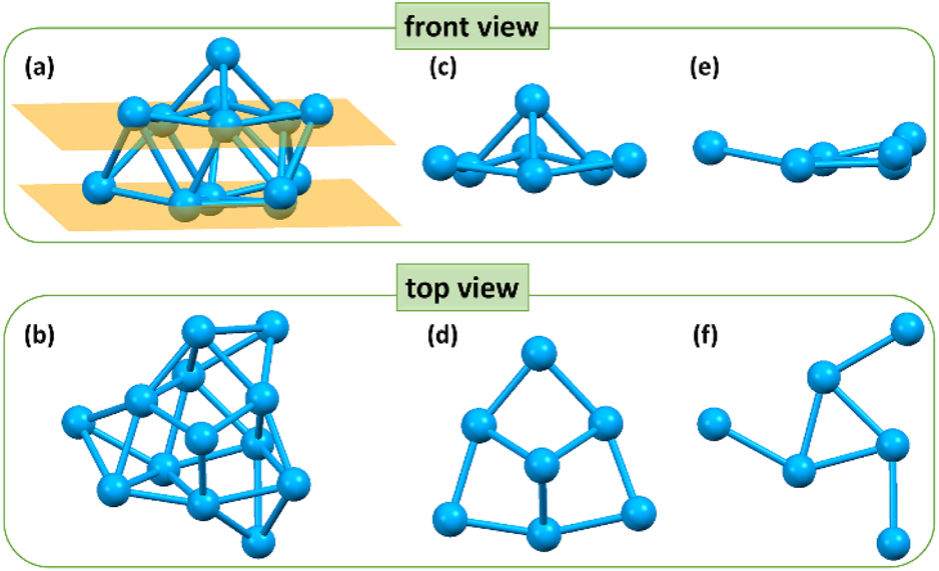
The front view and top view of (a and b) the skeleton structure, (c and d) first layer and (e and f) second layer of the Cu_13_H_10_(SR)_3_(PPh_3_)_7_ nanoclusters. Color code: blue, Cu.

Further structural investigation revealed that the Cu_13_ kernel could be divided into two layers (Fig. 2a and b). The first layer containing 7 Cu atoms looks like a hat from the front view, and is made up of four quadrilaterals from the top view (Fig. 2c and d). The second layer containing 6 Cu atoms is a propeller-like structural unit, which is a Cu_3_ triangle connected to three single Cu atoms from the top view (Fig. 2e and f). The average Cu–Cu length of the Cu_13_ nanoclusters is 2.57 Å with a range from 2.415 Å to 2.792 Å (Tables S2 and S3†), which is close to the Cu–Cu length of bulk Cu and lower than that of other reported Cu nanoclusters (Table S4†), indicating stronger Cu–Cu interactions.

**Fig. 3:**
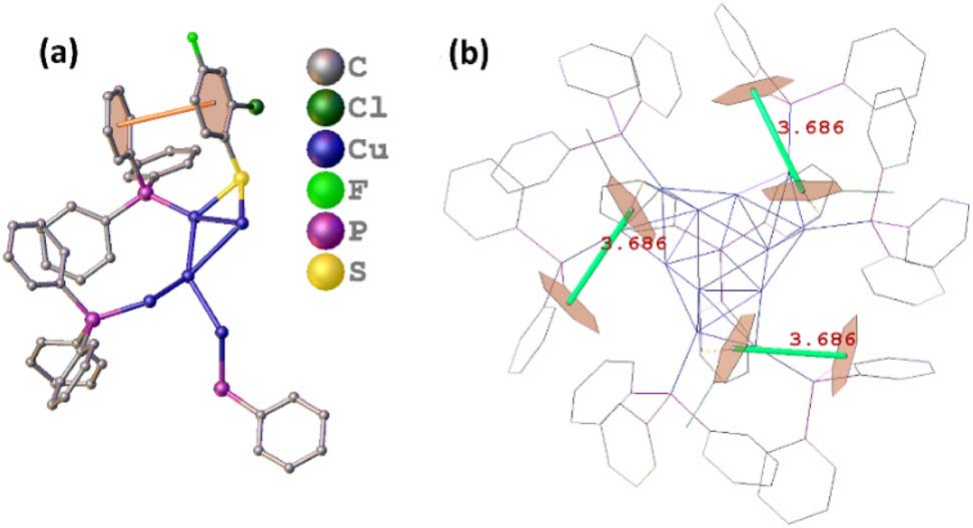
The π⋯π interactions between thiolates and PPh_3_ on the surface of the Cu_13_H_10_(SR)_3_(PPh_3_)_7_ nanoclusters shown in the asymmetric unit (a) and total structure (b).

With regard to the exterior protective monolayer of Cu_13_ nanoclusters, each of the seven PPh_3_ is directly connected to one Cu atom *via* a single Cu–P bond. One PPh_3_ is located on the C_3_ symmetry axis, and the other six PPh_3_, along with the three thiolates, are arranged symmetrically around the C_3_ axis (Fig. S3†). Each thiolate bonds with two Cu atoms in a low-coordinated μ_2_-η^1^, η^1^ bridging mode (Fig. S4†). The Cu–P and Cu–S bond length ranges are 2.229–2.240 Å (average: 2.237 Å) and 2.262–2.285 Å (average: 2.274 Å), respectively. The three Cu–S–Cu angles are 69.8° (Table S3†). It is worth mentioning that the weak inter-ligand interactions in the metal nanoclusters are important for the atomic construction of the surface structure and the unit cell packing arrangement. In the Cu_13_ nanoclusters, the intramolecular π⋯π interactions with a short distance (3.686 Å) exist between the benzene rings of the thiolates and PPh_3_ on the surface, resulting in the stable configuration (Fig. 3).

### Molecular formula and chemical state

**Fig. 4:**
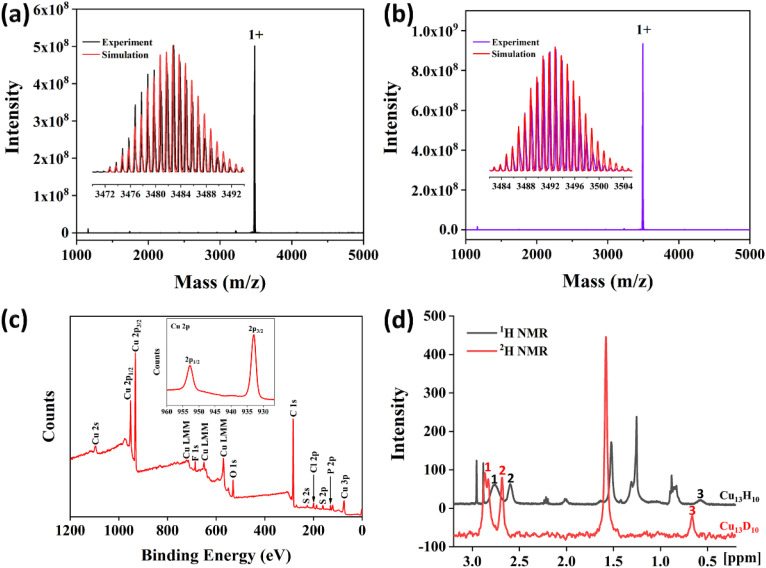
The ESI-MS of Cu_13_H_10_(SR)_3_(PPh_3_)_7_ (a) and Cu_13_D_10_(SR)_3_(PPh_3_)_7_ (b) nanoclusters in positive mode. Insets show the experimental and simulated isotopic patterns. (c) The X-ray photoelectron full spectra of Cu_13_H_10_(SR)_3_(PPh_3_)_7_ nanoclusters. Inset shows the XPS of Cu 2p. (d) ^1^H NMR of Cu_13_H_10_(SR)_3_(PPh_3_)_7_ (black) and ^2^H NMR of Cu_13_D_10_(SR)_3_(PPh_3_)_7_ nanoclusters (red).

To further identify the molecular formula, electrospray ionization mass spectroscopy (ESI-MS) of the Cu_13_ nanoclusters in positive mode was carried out using a Fourier transform ion cyclotron resonance mass spectrometer. As shown in Fig. 4a, a dominant peak with one positive charge was observed at *m*/*z* = 3482.7 in the mass spectrum. The inset spectra showed that the experimental value was highly consistent with the simulated isotopic distribution, corresponding to the formula [Cu_13_H_10_(SR)_3_(PPh_3_)_7_ + Cu^+^ + PPh_3_]^+^. To confirm the number of hydrogen atoms, the deuterated analogue was prepared using NaBD_4_ as the reducing agent. The ESI-MS of the deuterated analogue shows a prominent peak at *m*/*z* = 3492.7, which was attributed to the formula [Cu_13_D_10_(SR)_3_(PPh_3_)_7_ + Cu^+^ + PPh_3_]^+^, thus indicating the existence of 10 hydride ligands in the Cu_13_ nanoclusters (Fig. 4b).

X-ray photoelectron spectroscopy (XPS) was carried out to investigate the elemental composition and chemical states of Cu_13_ nanoclusters. XPS spectra confirmed the presence of the expected elements Cu, S, P, F, Cl, and C (Fig. 4c). The inset exhibits the binding energy for Cu 2p_1/2_ at 952.7 eV and Cu 2p_3/2_ at 932.9 eV in the Cu_13_ nanoclusters. However, it is difficult to identify the chemical state of Cu owing to the same Cu 2p_3/2_ binding energy (932.6 eV) being observed for both Cu^0^ and Cu^+^ (Cu_2_S) species.^4^ The three Cu LMM peaks can be observed in the XPS, and the smallest binding energy is 570.0 eV, which is same as that of Cu_2_O. The Cu LMM binding energy of Cu^0^ is 568.0 eV.^67^ Additionally, no satellite signal for Cu 2p_3/2_ from Cu(ii) was observed at approximately 943 eV in the XPS spectra of Cu_13_ nanoclusters. Therefore, the chemical state of Cu in the Cu_13_ nanoclusters is close to that of Cu(i), which is consistent with that of other Cu nanoclusters.^4,54^

Nuclear magnetic resonance (NMR) was performed to identify the chemical shifts of the hydrides in the Cu_13_ nanoclusters. In the ^1^H NMR spectra, Cu_13_ and Cu_13D_ nanoclusters exhibit similar peaks (Fig. S5†). The peaks at 8–6.4 ppm correspond to protons in the thiolates and PPh_3_ phenyl groups. The broadening of peaks between 8 and 6.4 ppm is ascribed to the differences in the environments of the ligands (Fig. S5†). Moreover, the Cu_13_ nanoclusters show three additional peaks centered at 2.76, 2.60 and 0.57 ppm in the ^1^H NMR spectrum, compared with the Cu_13D_ nanoclusters (Fig. S5† and 4d). The ratio of the areas of the three peaks at 2.76, 2.60 and 0.57 ppm is 6 : 3 : 1. Notably, the peak at 2.76 ppm is wide, probably because of the overlap of two peaks. To further determine the type and ratio of the hydrides, the ^2^H NMR of Cu_13_ and Cu_13D_ were measured. In the ^2^H NMR spectra, both the Cu_13_ and Cu_13D_ nanoclusters exhibit peaks at 7.26 and 1.57 ppm, corresponding to CDCl_3_ and D_2_O, respectively (Fig. S6†). However, the Cu_13D_ nanoclusters show four more peaks than the Cu_13_ nanoclusters; these peaks are centered at 2.86, 2.83, 2.68 and 0.66 ppm and correspond to the hydrides (Fig. S6† and 4d). The ratio of the areas of the four peaks at 2.86, 2.83, 2.68 and 0.66 ppm is 3 : 3 : 3 : 1. The two peaks at 2.86 and 2.83 ppm in the ^2^H NMR spectrum are so close that they obviously overlap. Accordingly, it can be inferred that the wide peak at 2.76 ppm in the ^1^H NMR spectrum is also due to the overlap of two peaks, considering the area and position of the peak (Table S5†). Therefore, combined with the ESI-MS results, the NMR data further demonstrates that it is reasonable that the Cu_13_ nanoclusters contain 10 hydrides, including four types of hydrides.

### Modeling the hydride positions

**Fig. 5:**
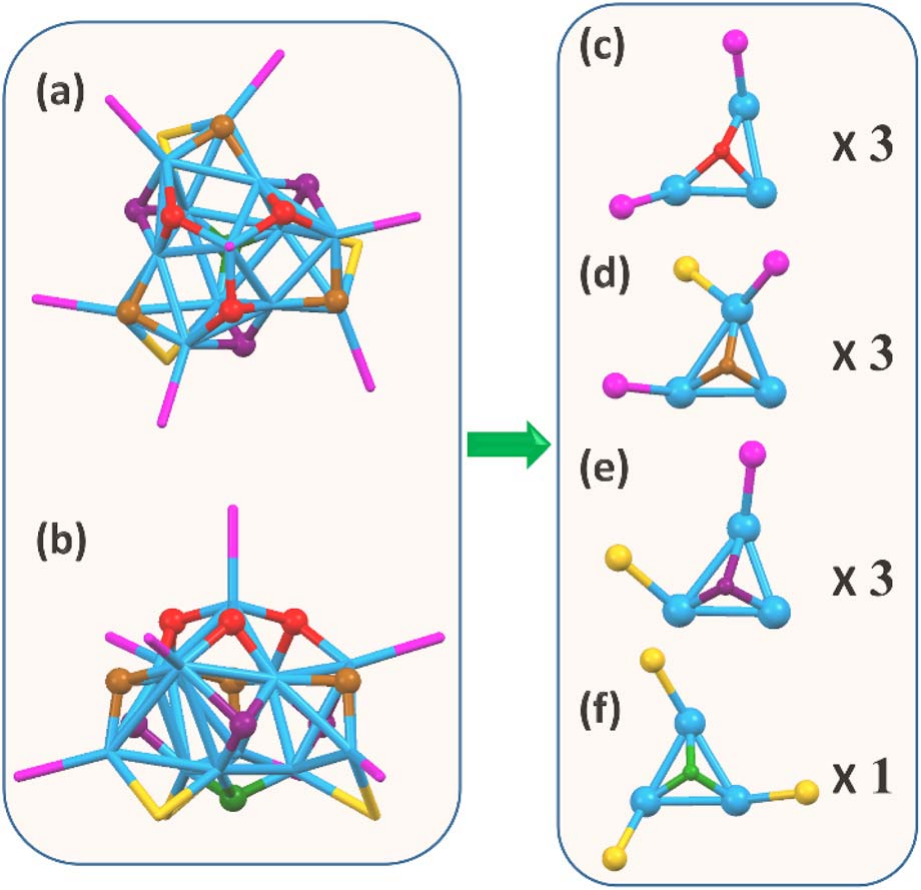
The top view (a) and side view (b) of the optimized structure for the Cu_13_H_10_(SR)_3_(PPh_3_)_7_ nanoclusters with the hydride sites in the kernel obtained from DFT calculations. (c–f) The hydrides binding with three Cu atoms can be seen to give four groups according to the triangular Cu_3_ coordination environment. Color code: blue, Cu; magenta, P; orange, S; red/brown/purple/green, μ_3_-H in the kernel. The carbon terminals of the ligands are omitted for clarity.

To determine the hydride positions in the Cu_13_ nanoclusters, DFT calculations were carried out to optimize the geometric structure of the Cu_13_ nanoclusters. Instead of the widely used simplified model in which the H–SR ligands in the thiolates are replaced by HS-CH_3_ to save computational resources, the full model with the entire Cu_13_ structure was used in this work to reproduce the effects of the ligands. This is especially important for our Cu_13_H_10_(SR)_3_(PPh_3_)_7_ nanoclusters, since intramolecular π⋯π interactions exist between the benzene rings of the thiolate and PPh_3_ ligands on the surface. The optimizations were based on the X-ray crystal structure (Fig. S7†), and the adopted strategy is similar to that in a previous report.^54^ First, only the hydrides were relaxed, and then both the hydrides and Cu were fully relaxed. Initial structures with μ_1_, μ_2_ and μ_3_ hydrides were tried, and all of them optimized to the same structure (Table S7†). The optimized structure of the Cu_13_ nanoclusters is in accordance with the crystallographic structure (Fig. S8†). The simulated hydride sites in the optimized structure of the Cu_13_ nanoclusters are shown in Fig. 5a and b. Each of the 10 hydrides was connected to three Cu atoms in a μ_3_ mode, and the hydrides could be categorized into four groups according to the triangular Cu_3_ coordination environment. The number of hydrides located at the top, middle and bottom is 3, (3 + 3) and 1, respectively. First, the three hydrides marked in red at the top are bound to a Cu_3_ triangle coordinated with two PPh_3_ (Fig. 5c). Second, the three hydrides marked in brown at the middle are connected to Cu_3_ triangles with one thiolate and two PPh_3_ (Fig. 5d). Third, the three hydrides marked in purple at the middle are connected to a Cu_3_ triangle with one thiolate and one PPh_3_ (Fig. 5e). Fourth, the only hydride marked in green at the bottom is linked to a Cu_3_ triangle with three thiolates (Fig. 5f). Of note, the two types of hydrides located at the middle have similar coordination environments, so they show nearly the same chemical shifts and their peaks overlap in the NMR spectra. The types and number of hydrides from the optimized structure are in good agreement with the NMR results.

### Optical properties

**Fig. 6:**
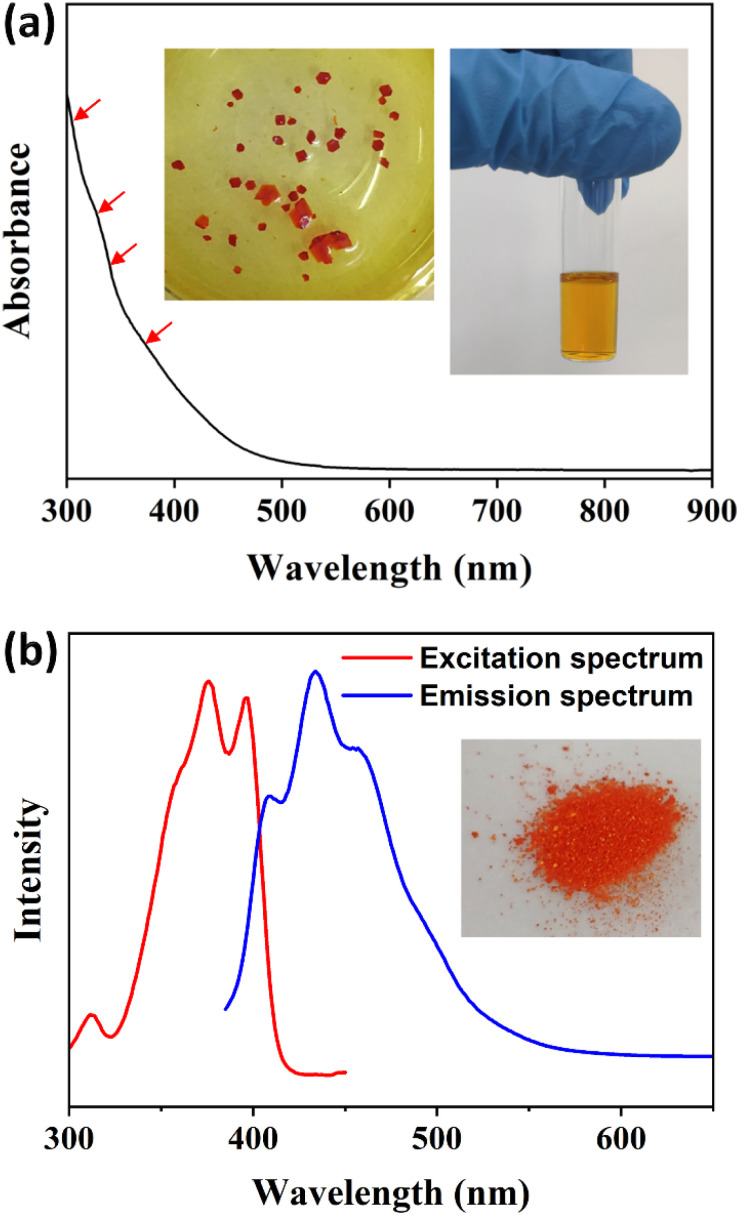
(a) The UV-vis absorption spectrum of Cu_13_H_10_(SR)_3_(PPh_3_)_7_ nanoclusters. The insets show the crystals and solution of the nanoclusters. (b) The excitation (*λ*_em_ = 460 nm) and emission (*λ*_ex_ = 375 nm) spectra of Cu_13_H_10_(SR)_3_(PPh_3_)_7_ nanoclusters. The inset shows solid nanoclusters obtained *via* large-scale SMPS.

The steady-state UV-vis absorption spectrum of the Cu_13_ nanoclusters exhibits four weak broad peaks at 305, 325, 340 and 360 nm (Fig. 6a). The absorption peaks are not entirely obvious, which is a common phenomenon in Cu nanoclusters.^4,33,54,68^ The UV-vis absorption spectrum was calculated using the time-dependent DFT method based on the entire structure of the Cu_13_ nanoclusters, and a decay curve can be observed (Fig. S9†). Actually, several absorption peaks at 300.9, 304.4, 311.8, 326.3, 328.2, 340.5, 342.4, 356.0 and 364.1 nm were predicted according to the oscillator strength (Fig. S9†). In summary, the calculated absorption peaks mainly lie in four approximate bands: 300–304 nm, 326–328 nm, 340–342 nm and 356–364 nm, which is in good agreement with the experimental spectrum. The Kohn–Sham molecular orbitals of the Cu_13_ nanoclusters are shown in Fig. S10.† The highest occupied molecular orbital (HOMO) and the lowest unoccupied molecular orbital (LUMO) are mainly composed of Cu and S atoms. Based on the maximum contribution to the absorption, the absorption peaks at 311.8, 328.2, 356.0 and 364.1 nm are attributed to the transition from Cu (3d) to Cu (4p). The other peaks arise from the transition between Cu (3d) and ligand π* (Table S6†).

The photoluminescence properties of the Cu_13_ nanoclusters were studied *via* fluorescence spectroscopy. As shown in Fig. 6b, the excitation spectrum shows three apparent peaks at 312, 375 and 396 nm with a shoulder at approximately 360 nm. Under excitation at 375 nm, the unique emission spectrum exhibits three obvious peaks at 408, 434 and 458 nm, respectively. Furthermore, we carried out fluorescence spectroscopy for the Cu_13_ nanoclusters at low temperatures (Fig. S11†). As the temperature decreases, the intensity of the photoluminescence increases. The blue emission of the Cu_13_ nanoclusters is remarkable, and will probably have promising applications such as cell labelling.^69–71^ The photoluminescence might originate from the LMCT (ligand-to-metal charge transfer) process. The fluorescence is attributed to the electronic transition from the LUMO to the HOMO, and is mainly caused by charge transfer between the surface ligands with electron-donating capability and the Cu atoms with electron-withdrawing capability in the kernel.^72,73^

**Fig. 7:**
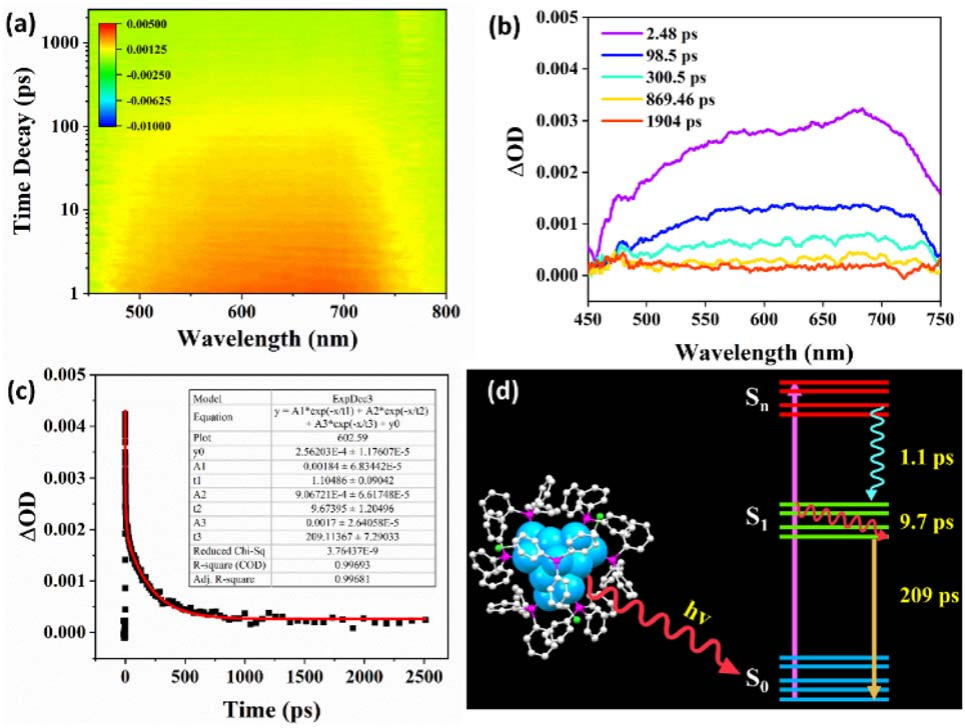
(a) Transient absorption spectra data map of Cu_13_H_10_(SR)_3_(PPh_3_)_7_ pumped at 360 nm. (b) Transient absorption spectra of Cu_13_H_10_(SR)_3_(PPh_3_)_7_ at various times after excitation. (c) Kinetic traces monitored at around 600 nm (black) and curve fitting through the exponential equation (red). (d) The schematic illustration of the proposed relaxation dynamics of the Cu_13_H_10_(SR)_3_(PPh_3_)_7_ nanoclusters.

Exploring the excited-state dynamics of metal nanoclusters is essential to promote their practical applications. Femtosecond transient absorption spectroscopy was employed to study the excited-state properties of the Cu_13_ nanoclusters. The data map of the transient absorption spectrum as a function of the time delay after a 360 nm pump pulse is shown in Fig. 7a. It displays broad excited-state absorption (ESA) peaks at approximately 580 and 680 nm. Fig. 7b shows the transient absorption at different decay times, and the transient absorption signal decays almost to zero within 869.46 ps. Global fitting with a three-exponential-decay equation was applied to fit the time profile to analyze the ultrafast relaxation dynamics (Fig. 7c). To acquire good fitting quality, fitting the population dynamics of the transient absorption required three decay components: 1.1, 9.7, and 209 ps, respectively. This is similar to other metal nanoclusters.^68,74^Fig. 7d exhibits a schematic illustration of the proposed relaxation dynamics of the Cu_13_ nanoclusters. The ultrafast 1.1 ps component should be ascribed to the internal conversion from the S_*n*_ to the S_1_ state. The 9.7 ps component can be attributed to the structural relaxation of vibrational cooling from hot S_1_ to S_1_, while the 209 ps component corresponds to the radiative relaxation from S_1_ to the ground state S_0_ (Fig. 7d). Revealing the excited-state relaxation dynamics of Cu_13_ nanoclusters is favorable for its applications in energy conversion and photoelectric applications.

## Conclusions

In summary, a novel synthetic strategy named solvent-mediated precipitating synthesis (SMPS) was developed to prepare high-purity Cu_13_H_10_(SR)_3_(PPh_3_)_7_ nanoclusters with high yield. The Cu_13_ nanoclusters comprise a Cu_13_ kernel and a monolayer of ligands including hydride, thiolate and PPh_3_. The aesthetic Cu_13_ kernel consists of four rare vertex-sharing tetrahedrons, and the ligands are arranged symmetrically around the C_3_ symmetry axis. The intramolecular π⋯π interactions between thiolates and PPh_3_ on the surface are conducive to the stable configuration. The number, type and location of the 10 hydrides were revealed comprehensively. Furthermore, the Cu_13_ nanoclusters exhibit unique optical absorbance and photoluminescence. The transient absorption spectra revealed the ultrafast relaxation dynamics behaviors of internal conversion, structural relaxation and radiative relaxation of the Cu_13_ nanoclusters. This work provides an efficient strategy to synthesize a novel Cu nanocluster.

## Data availability

We have provided the experimental and computational data in the ESI.†

## Author contributions

X. L. conceived the project, designed the synthesis, and analyzed data. J. T., C. Z. and Y. Y. performed the measurements. L. W. and R. W. tested the ESI-MS. F. H. finished the DFT calculations. C. L. and J. H. conceived the project and provided the guidance. The manuscript was written through contributions of all authors. All authors have given approval to the final version of the manuscript.

## Conflicts of interest

There are no conflicts to declare.

## Supplementary Material

SC-014-D2SC06099J-s001

SC-014-D2SC06099J-s002

## References

[cit1] Dong C., Huang R. W., Chen C., Chen J., Nematulloev S., Guo X., Ghosh A., Alamer B., Hedhili M. N., Isimjan T. T., Han Y., Mohammed O. F., Bakr O. M. (2021). J. Am. Chem. Soc..

[cit2] Lee S., Bootharaju M. S., Deng G., Malola S., Hakkinen H., Zheng N., Hyeon T. (2021). J. Am. Chem. Soc..

[cit3] Zhang L. L. M., Zhou G. D., Zhou G. Q., Lee H. K., Zhao N., Prezhdo O. V., Mak T. C. W. (2019). Chem. Sci..

[cit4] Nematulloev S., Huang R. W., Yin J., Shkurenko A., Dong C., Ghosh A., Alamer B., Naphade R., Hedhili M. N., Maity P., Eddaoudi M., Mohammed O. F., Bakr O. M. (2021). Small.

[cit5] Han B.-L., Liu Z., Feng L., Wang Z., Gupta R. K., Aikens C. M., Tung C. H., Sun D. (2020). J. Am. Chem. Soc..

[cit6] Li J. C., Kuang Y., Meng Y. T., Tian X., Hung W. H., Zhang X., Li A. W., Xu M. Q., Zhou W., Ku C. S., Chiang C. Y., Zhu G. Z., Guo J. Y., Sun X. M., Dai H. J. (2020). J. Am. Chem. Soc..

[cit7] Wang Y. H., Xu A., Wang Z. Y., Huang L. S., Li J., Li F. W., Wicks J., Luo M. C., Nam D. H., Tan C. S., Ding Y., Wu J. W., Lum Y. W., Dinh C. T., Sinton D., Zheng G. F., Sargent E. H. (2020). J. Am. Chem. Soc..

[cit8] Chen C. A., Chen S. C., Shiddiky M. J. A., Chen C. F., Wu K. C. W. (2020). Chem. Rec..

[cit9] Peng F., Sun Y., Lu Y., Yu W. W., Ge M. Y., Shi J. C., Cong R., Hao J. M., Dai N. (2020). Nanomaterials.

[cit10] Yang P. P., Zhang X. L., Gao F. Y., Zheng Y. R., Niu Z. Z., Yu X. X., Liu R., Wu Z. Z., Qin S., Chi L. P., Duan Y., Ma T., Zheng X. S., Zhu J. F., Wang H. J., Gao M. R., Yu S. H. (2020). J. Am. Chem. Soc..

[cit11] Zhang L., Li X. X., Lang Z. L., Liu Y., Liu J., Yuan L., Lu W. Y., Xia Y. S., Dong L. Z., Yuan D. Q., Lan Y. Q. (2021). J. Am. Chem. Soc..

[cit12] Yao Q., Wu Z., Liu Z., Lin Y., Yuan X., Xie J. (2021). Chem. Sci..

[cit13] Sun C., Teo B. K., Deng C., Lin J., Luo G. G., Tung C. H., Sun D. (2021). Coord. Chem. Rev..

[cit14] Zhang W. J., Liu Z., Song K. P., Aikens C. M., Zhang S. S., Wang Z., Tung C. H., Sun D. (2021). Angew. Chem., Int. Ed..

[cit15] Li Y., Jin R. (2020). J. Am. Chem. Soc..

[cit16] Zhou Y., Liao L., Zhuang S., Zhao Y., Gan Z., Gu W., Li J., Deng H., Xia N., Wu Z. (2021). Angew. Chem., Int. Ed..

[cit17] Heaven M. W., Dass A., White P. S., Holt K. M., Murray R. W. (2008). J. Am. Chem. Soc..

[cit18] Zhu M., Aikens C. M., Hollander F. J., Schatz G. C., Jin R. (2008). J. Am. Chem. Soc..

[cit19] Cao Y., Fung V., Yao Q., Chen T., Zang S., Jiang D. E., Xie J. (2020). Nat. Commun..

[cit20] Zhao Y., Zhuang S., Liao L., Wang C., Xia N., Gan Z., Gu W., Li J., Deng H., Wu Z. (2020). J. Am. Chem. Soc..

[cit21] Qian H. F., Zhu Y., Jin R. C. (2010). J. Am. Chem. Soc..

[cit22] Zeng C., Chen Y., Liu C., Nobusada K., Rosi N. L., Jin R. (2015). Sci. Adv..

[cit23] Gan Z., Liu Y., Wang L., Jiang S., Xia N., Yan Z., Wu X., Zhang J., Gu W., He L., Dong J., Ma X., Kim J., Wu Z., Xu Y., Li Y., Wu Z. (2020). Nat. Commun..

[cit24] Gan Z. B., Chen J. S., Liao L. W., Zhang H. W., Wu Z. K. (2018). J. Phys. Chem. Lett..

[cit25] Liao L., Chen J., Wang C., Zhuang S., Yan N., Yao C., Xia N., Li L., Bao X., Wu Z. (2016). Chem. Commun..

[cit26] Zeng C. J., Liu C., Chen Y. X., Rosi N. L., Jin R. C. (2016). J. Am. Chem. Soc..

[cit27] Wang J. Q., Shi S., He R. L., Yuan S. F., Yang G. Y., Liang G. J., Wang Q. M. (2020). J. Am. Chem. Soc..

[cit28] Sakthivel N. A., Theivendran S., Ganeshraj V., Oliver A. G., Dass A. (2017). J. Am. Chem. Soc..

[cit29] Joshi C. P., Bootharaju M. S., Alhilaly M. J., Bakr O. M. (2015). J. Am. Chem. Soc..

[cit30] Yang H., Yan J., Wang Y., Deng G., Su H., Zhao X., Xu C., Teo B. K., Zheng N. (2017). J. Am. Chem. Soc..

[cit31] Ren L., Yuan P., Su H., Malola S., Lin S., Tang Z., Teo B. K., Hakkinen H., Zheng L., Zheng N. (2017). J. Am. Chem. Soc..

[cit32] Yang H. Y., Wang Y., Chen X., Zhao X. J., Gu L., Huang H. Q., Yan J. Z., Xu C. F., Li G., Wu J. C., Edwards A. J., Dittrich B., Tang Z. C., Wang D. D., Lehtovaara L., Hakkinen H., Zheng N. F. (2016). Nat. Commun..

[cit33] Li H., Zhai H., Zhou C., Song Y., Ke F., Xu W. W., Zhu M. (2020). J. Phys. Chem. Lett..

[cit34] Santiago-Gonzalez B., Monguzzi A., Capitani C., Prato M., Santambrogio C., Meinardi F., Brovelli S. (2018). Angew. Chem., Int. Ed..

[cit35] Chakrahari K. K., Silalahi R. P. B., Liao J. H., Kahlal S., Liu Y. C., Lee J. F., Chiang M. H., Saillard J. Y., Liu C. W. (2018). Chem. Sci..

[cit36] Ke F., Song Y. B., Li H., Zhou C. J., Du Y. X., Zhu M. Z. (2019). Dalton Trans..

[cit37] Chakrahari K. K., Liao J. H., Kahlal S., Liu Y. C., Chiang M. H., Saillard J. Y., Liu C. W. (2016). Angew. Chem., Int. Ed..

[cit38] Rodriguez-Kessler P. L., Rojas-Poblete M., Munoz-Castro A. (2021). Phys. Chem. Chem. Phys..

[cit39] Chen Z., Wang Y., Liu L., Zhang Z., Liang F. (2012). Chem. Commun..

[cit40] Nguyen T. A., Jones Z. R., Goldsmith B. R., Buratto W. R., Wu G., Scott S. L., Hayton T. W. (2015). J. Am. Chem. Soc..

[cit41] Cook A. W., Jones Z. R., Wu G., Scott S. L., Hayton T. W. (2018). J. Am. Chem. Soc..

[cit42] Dhayal R. S., Liao J. H., Lin Y.-R., Liao P.-K., Kahlal S., Saillard J. Y., Liu C. W. (2013). J. Am. Chem. Soc..

[cit43] Dhayal R. S., Liao J. H., Wang X., Liu Y. C., Chiang M. H., Kahlal S., Saillard J. Y., Liu C. W. (2015). Angew. Chem., Int. Ed..

[cit44] Huang R.-W., Yin J., Dong C., Maity P., Hedhili M. N., Nematulloev S., Alamer B., Ghosh A., Mohammed O. F., Bakr O. M. (2020). ACS Mater. Lett..

[cit45] Maity S., Bain D., Chakraborty S., Kolay S., Patra A. (2020). ACS Sustainable Chem. Eng..

[cit46] Li F., Tang Q. (2020). J. Catal..

[cit47] Chen A., Kang X., Jin S., Du W., Wang S., Zhu M. (2019). J. Phys. Chem. Lett..

[cit48] Edwards A. J., Dhayal R. S., Liao P. K., Liao J. H., Chiang M. H., Piltz R. O., Kahlal S., Saillard J. Y., Liu C. W. (2014). Angew. Chem., Int. Ed..

[cit49] Dhayal R. S., Liao J. H., Kahlal S., Wang X., Liu Y. C., Chiang M. H., van Zyl W. E., Saillard J. Y., Liu C. W. (2015). Chem.–Eur. J..

[cit50] Lee S., Bootharaju M. S., Deng G., Malola S., Baek W., Hakkinen H., Zheng N., Hyeon T. (2020). J. Am. Chem. Soc..

[cit51] Yuan P., Chen R. H., Zhang X. M., Chen F. J., Yan J. Z., Sun C. F., Ou D. H., Peng J., Lin S. C., Tang Z. C., Teo B. K., Zheng L. S., Zheng N. F. (2019). Angew. Chem., Int. Ed..

[cit52] Qu M., Zhang F. Q., Wang D. H., Li H., Hou J. J., Zhang X. M. (2020). Angew. Chem., Int. Ed..

[cit53] Ghosh A., Huang R.-W., Alamer B., Abou-Hamad E., Hedhili M. N., Mohammed O. F., Bakr O. M. (2019). ACS Mater. Lett..

[cit54] Huang R. W., Yin J., Dong C., Ghosh A., Alhilaly M. J., Dong X., Hedhili M. N., Abou-Hamad E., Alamer B., Nematulloev S., Han Y., Mohammed O. F., Bakr O. M. (2020). J. Am. Chem. Soc..

[cit55] Brust M., Walker M., Bethell D., Schiffrin D. J., Whyman R. (1994). J. Chem. Soc., Chem. Commun..

[cit56] Wei W., Lu Y., Chen W., Chen S. (2011). J. Am. Chem. Soc..

[cit57] Baghdasaryan A., Burgi T. (2021). Nanoscale.

[cit58] Liu X., Astruc D. (2018). Coord. Chem. Rev..

[cit59] Sheldrick G. M. (2008). Acta Crystallogr., Sect. A: Found. Crystallogr..

[cit60] Dolomanov O. V., Bourhis L. J., Gildea R. J., Howard J. A. K., Puschmann H. (2009). J. Appl. Crystallogr..

[cit61] Sheldrick G. M. (2015). Acta Crystallogr., Sect. C: Struct. Chem..

[cit62] Frisch M. J. , Trucks G. W., Schlegel H. B., Scuseria G. E., Robb M. A., Cheeseman J. R., Scalmani G., Barone V., Petersson G. A. and Nakatsuji H., *et al.*, Gaussian 16 program package, Gaussian, Inc., Wallingford CT, 2016

[cit63] Chai J. D., Head-Gordon M. (2008). Phys. Chem. Chem. Phys..

[cit64] Weigend F., Ahlrichs R. (2005). Phys. Chem. Chem. Phys..

[cit65] Wolinski K., Hinton J. F., Pulay P. (1990). J. Am. Chem. Soc..

[cit66] Lu T., Chen F. W. (2012). J. Comput. Chem..

[cit67] Platzman I., Brener R., Haick H., Tannenbaum R. (2008). J. Phys. Chem. C.

[cit68] Maity S., Bain D., Bhattacharyya K., Das S., Bera R., Jana B., Paramanik B., Datta A., Patra A. (2017). J. Phys. Chem. C.

[cit69] Ghosh R., Sahoo A. K., Ghosh S. S., Paul A., Chattopadhyay A. (2014). ACS Appl. Mater. Interfaces.

[cit70] Wang Z. G., Chen B. K., Rogach A. L. (2017). Nanoscale Horiz..

[cit71] Shi Y. e., Ma J., Feng A., Wang Z., Rogach A. L. (2021). Aggregate.

[cit72] Kang X., Zhu M. (2019). Chem. Soc. Rev..

[cit73] Kang X., Wang S. X., Song Y. B., Jin S., Sun G. D., Yu H. Z., Zhu M. Z. (2016). Angew. Chem., Int. Ed..

[cit74] Higaki T., Liu C., Zhou M., Luo T. Y., Rosi N. L., Jin R. C. (2017). J. Am. Chem. Soc..

