# Supplementary material for: Solvent-mediated precipitating synthesis and optical properties of polyhydrido Cu13 nanoclusters with four vertex-sharing tetrahedrons
Source: Chem Sci. 2022 Dec 20;14(4):994–1002. doi: 10.1039/d2sc06099j (PMC9890966; doi:10.1039/d2sc06099j)
Supplement: SC-014-D2SC06099J-s001 [file SC-014-D2SC06099J-s001.pdf]

Electronic Supplementary Material (ESI) for Chemical Science.
This journal is © The Royal Society of Chemistry 2022

Supplementary Information

Solvent Mediated Precipitating Synthesis and Optical Properties of Polyhydrido Cu_{13} Nanoclusters with Four Vertex-sharing Tetrahedrons

Xinzhang Lin,[ad] Jie Tang,[ad] Chenyu Zhu,[a] Li Wang,[b] Yang Yang,[ad] Ren`an Wu,[b] Hongjun Fan,*[c] Chao Liu,*[a] and Jiahui Huang*[a]

[a] Dalian National Laboratory for Clean Energy, Dalian Institute of Chemical Physics, Chinese Academy of Sciences, Dalian 116023, China

[b] Laboratory of High-Resolution Mass Spectrometry Technologies, Dalian Institute of Chemical Physics, Chinese Academy of Sciences, Dalian 116023, China

[c] State Key Laboratory of Molecular Reaction Dynamics, Dalian Institute of Chemical Physics, Chinese Academy of Sciences, Dalian 116023, China

[d] University of Chinese Academy of Sciences, Beijing 100049, China

* Corresponding to: fanhj@dicp.ac.cn; chaoliu@dicp.ac.cn; jiahuihuang@dicp.ac.cn

1. Figures

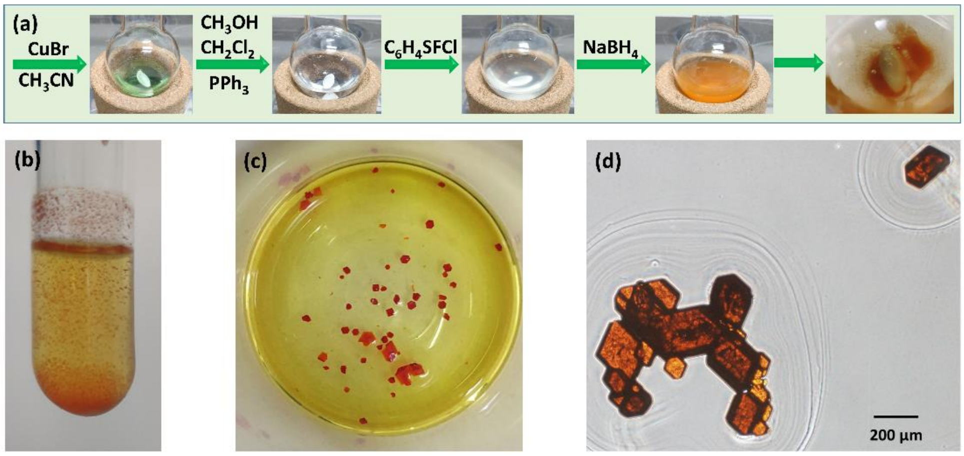

Figure S1. The schematic illustration of the SMPS synthetic route (a), electronic photos of single crystals shot on MI 8 AI dual camera (b-c) and microscope (d).

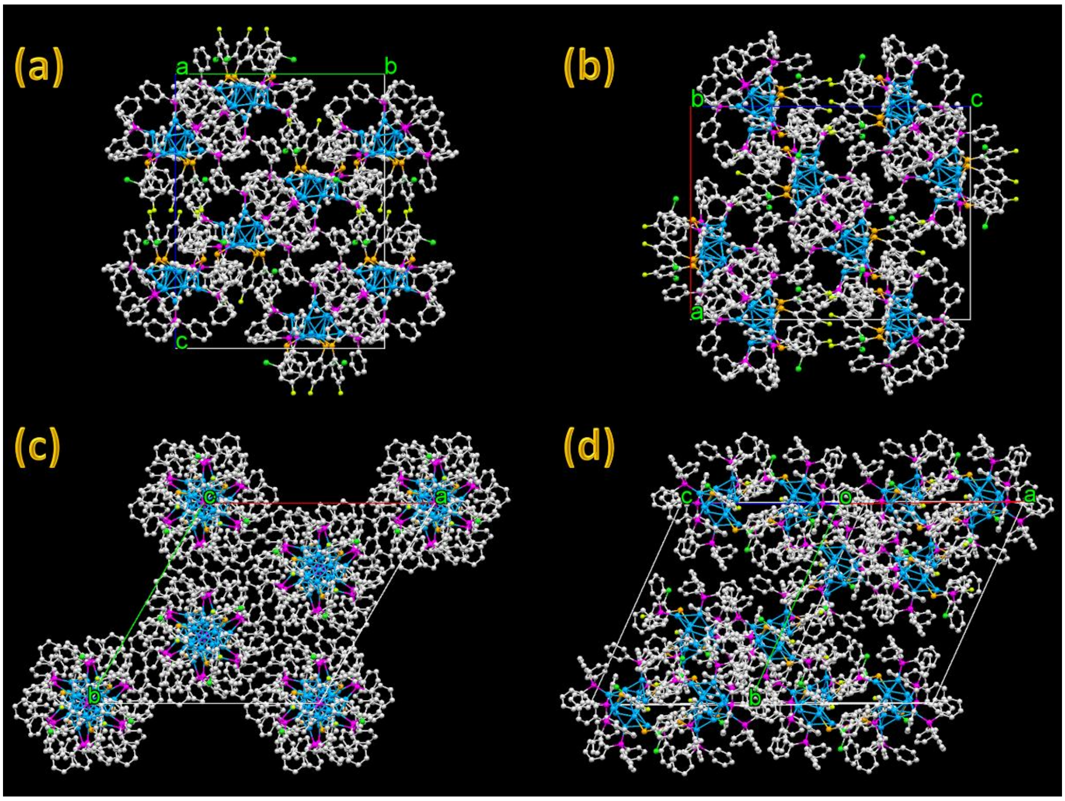

Figure S2. The packing arrangement of twelve $Cu_{13}H_{10}(SR)_3(PPh_3)_7$ nanoclusters in a unit cell from a, b, c directions, respectively. The hydrogen atoms are omitted for clarity. Color code: blue, Ag; orange, S; magenta, P; green, Cl; yellow, F; grey, C.

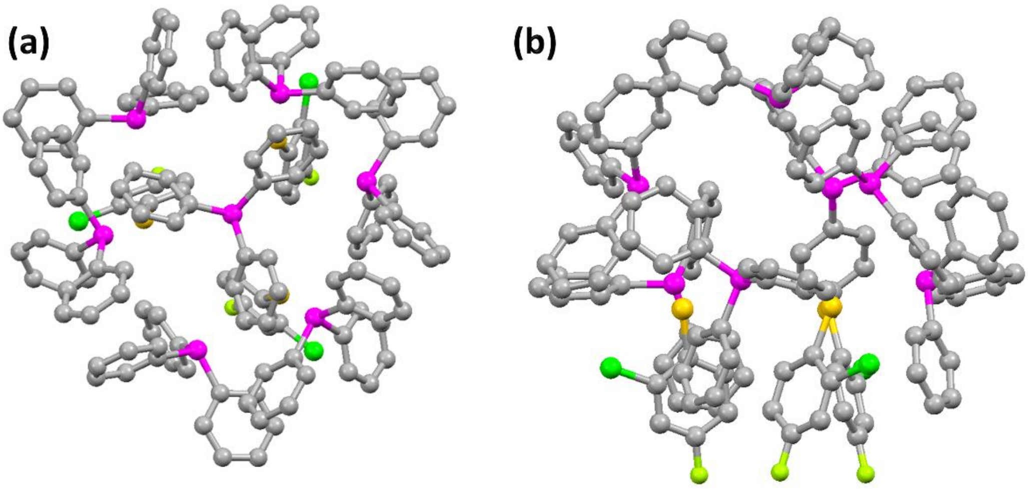

Figure S3. The top view (a) and side view (b) of the surface protected mono-layer of $Cu_{13}H_{10}(SR)_3(PPh_3)_7$ nanoclusters. One PPh_3 locates in the C_3 symmetry axis, and other 6 PPh_3 along with 3 thiolates are arranged symmetrically around the C_3 axis.

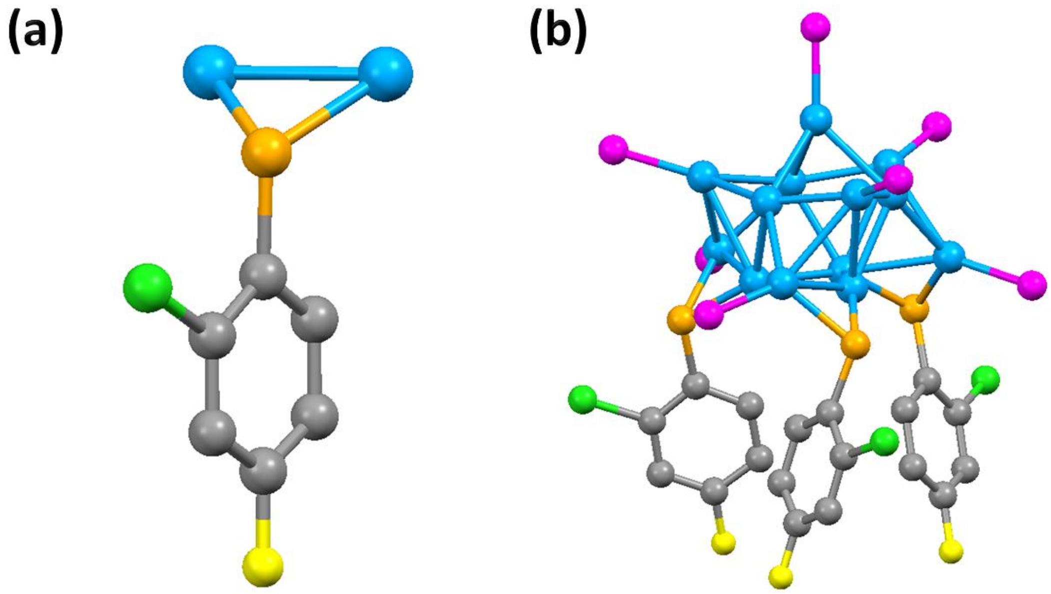

Figure S4. The S-Ag connecting mode in $Cu_{13}H_{10}(SR)_3(PPh_3)_7$ nanoclusters. Color code: blue, Ag; orange, S; magenta, P; green, Cl; yellow, F; grey, C.

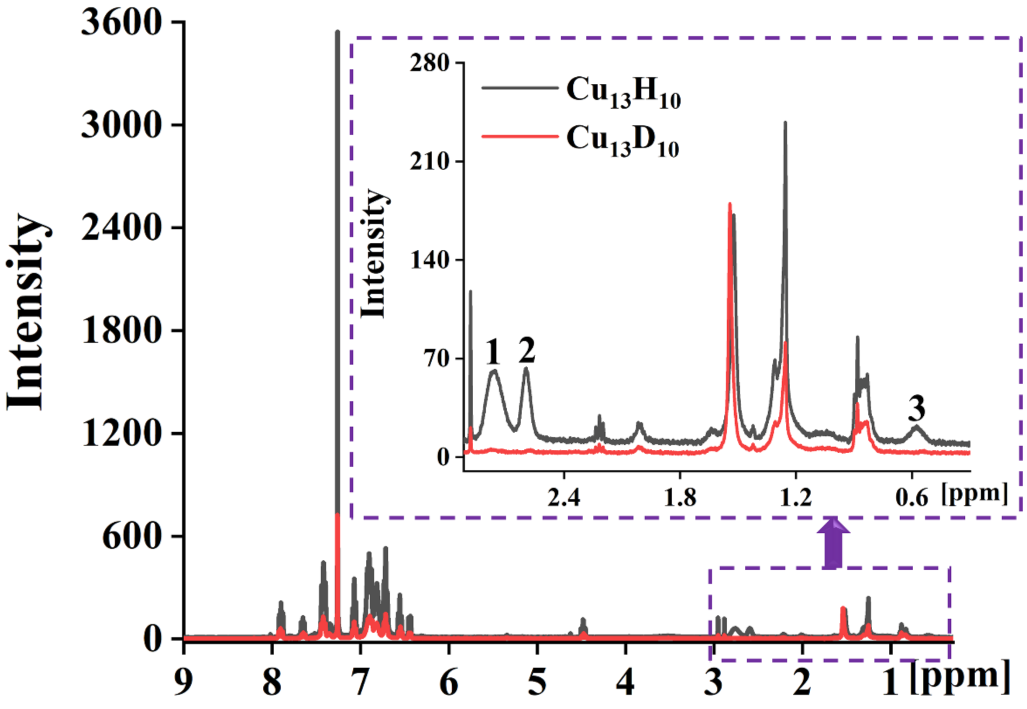

Figure S5. ^{1}H NMR of $Cu_{13}H_{10}(SR)_3(PPh_3)_7$ and $Cu_{13}D_{10}(SR)_3(PPh_3)_7$ nanoclusters.

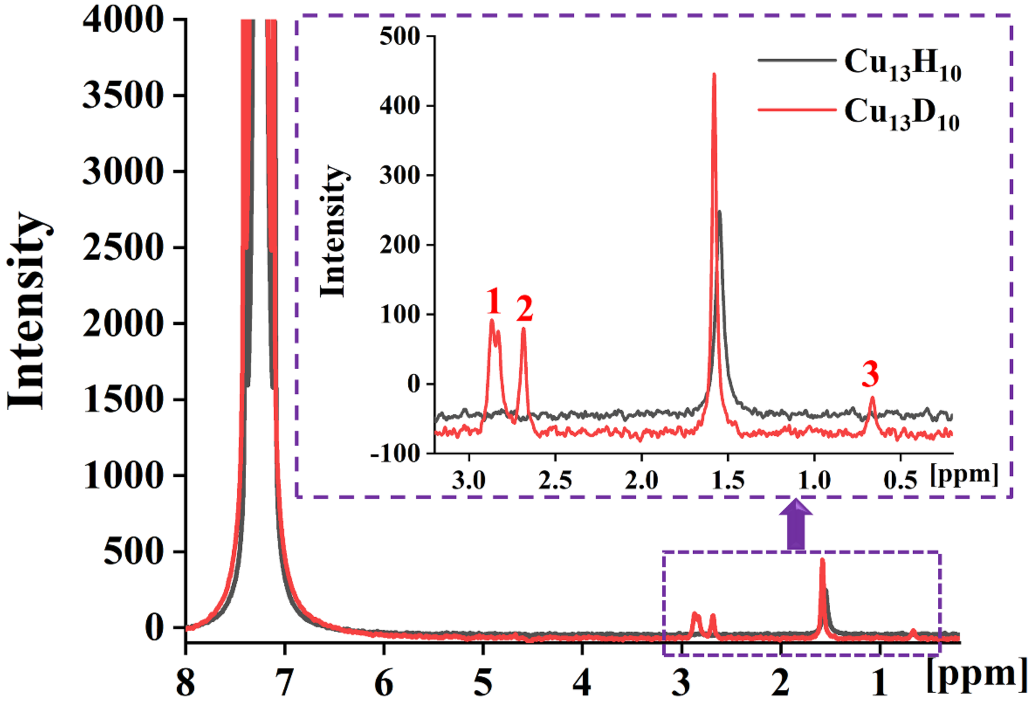

Figure S6. ^{2}H NMR of $Cu_{13}H_{10}(SR)_3(PPh_3)_7$ and $Cu_{13}D_{10}(SR)_3(PPh_3)_7$ nanoclusters.

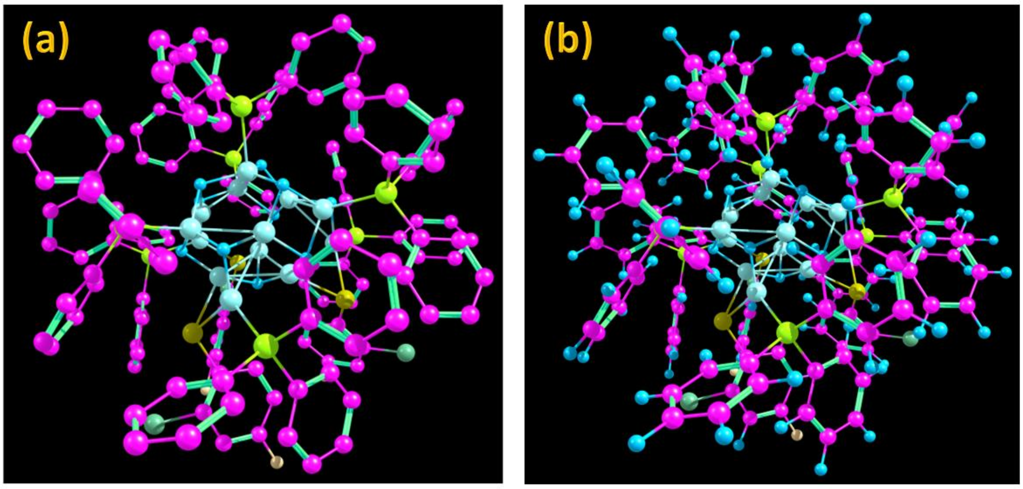

Figure S7. The optimized structure of $Cu_{13}H_{10}(SR)_3(PPh_3)_7$ nanoclusters. (a) Only H atoms bound to Cu atoms are shown. (b) All H atoms are shown.

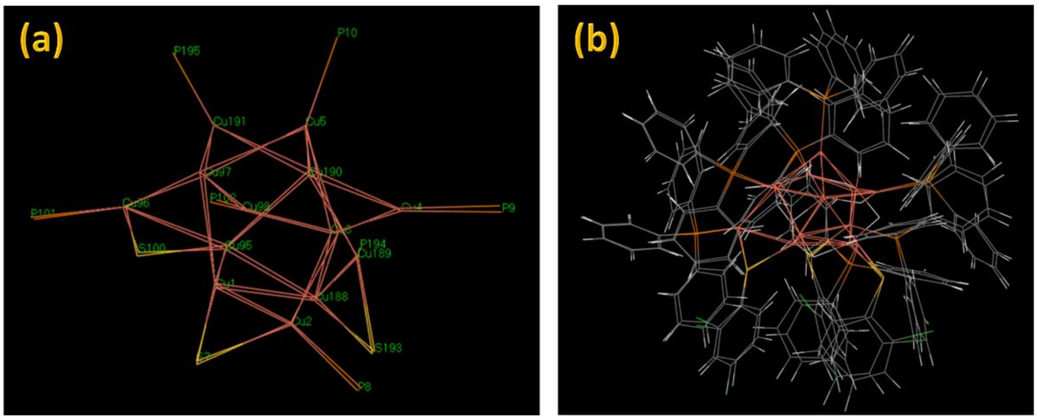

Figure S8. (a) Skeleton structure and (b) total structure for structural comparison between experimental and optimized $Cu_{13}H_{10}(SR)_3(PPh_3)_7$ nanoclusters.

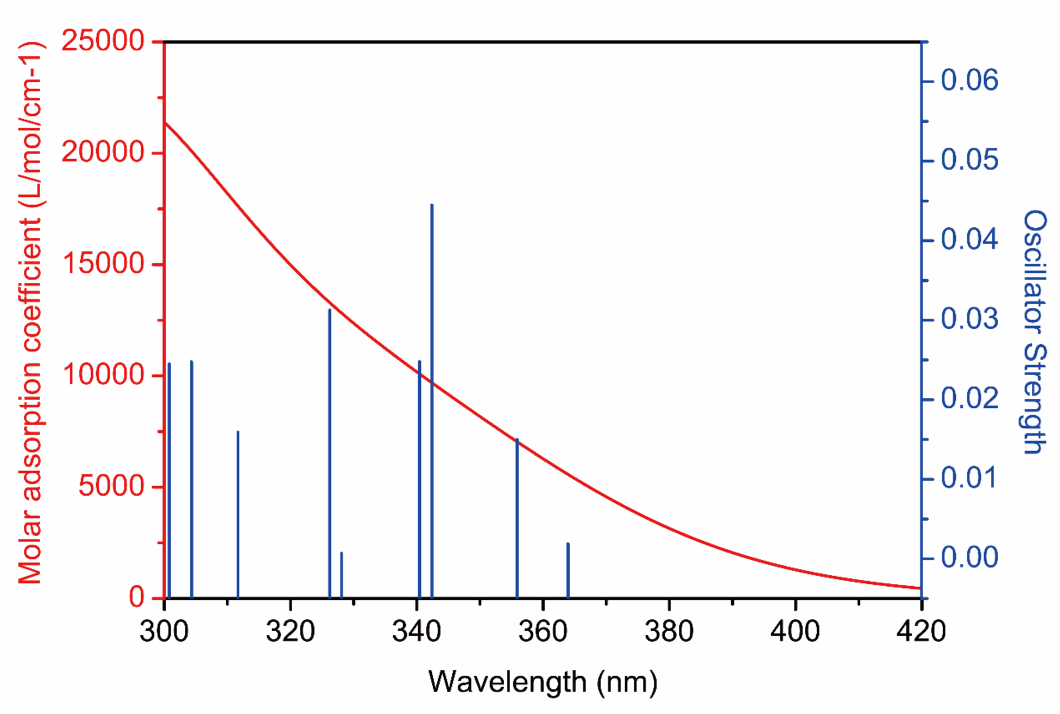

Figure S9. The calculated UV-vis absorption spectrum of $Cu_{13}H_{10}(SR)_3(PPh_3)_7$ nanoclusters. (Note: We show the simulated spectra in order to confirm that the calculated peaks are able to reproduce the experimental spectrum with certain half-width broadening Gaussian (40 nm), therefore are not conflicted with the experimental spectrum. Please note that simulated spectra strongly depend on the half-width used in the simulation. For example, with a smaller half-width (<25 nm) the simulated spectra will have a neat band with maximum around 340 nm, therefore the calculated adsorptions do not directly predict the experimental spectra)

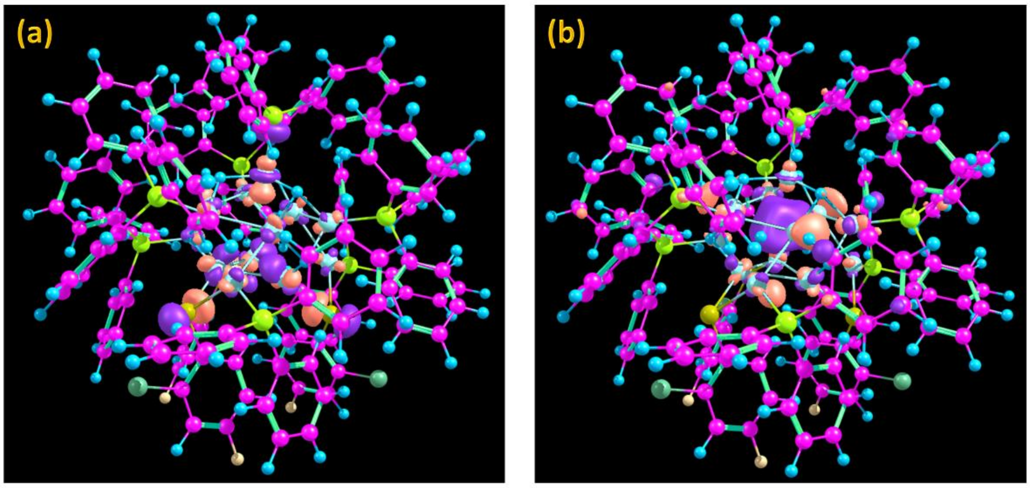

Figure S10. HOMO and LUMO of $Cu_{13}H_{10}(SR)_3(PPh_3)_7$ nanoclusters.

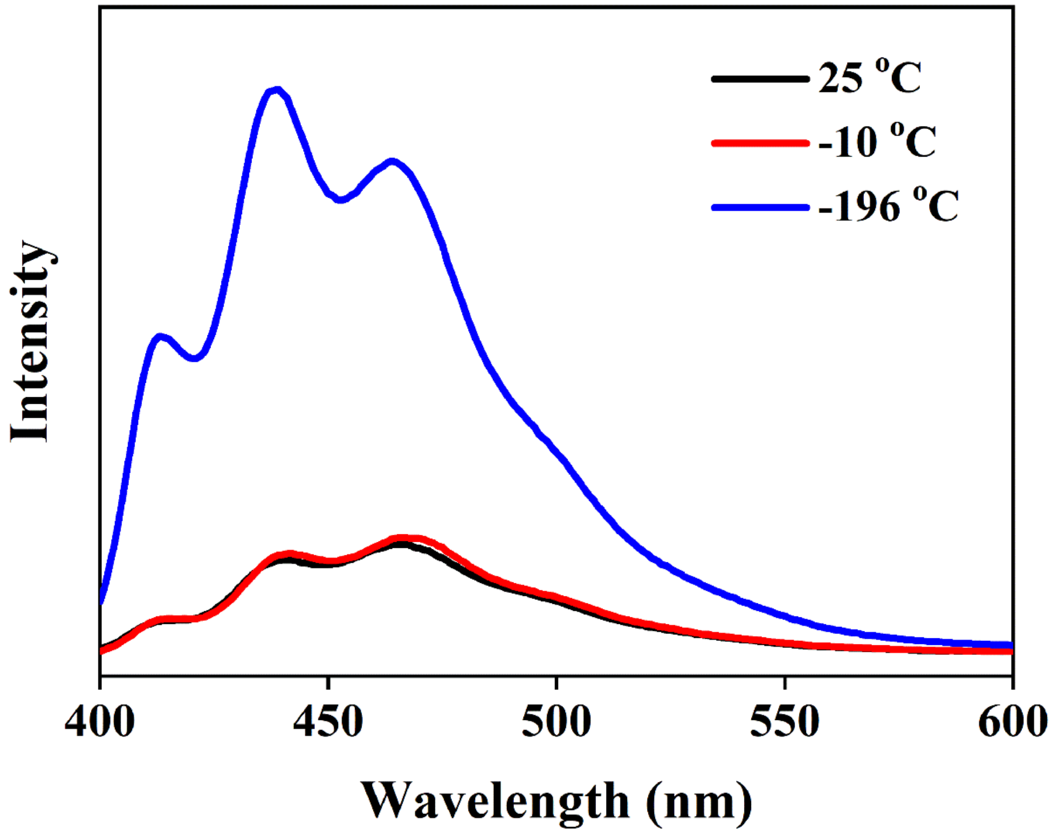

Figure S11. The fluorescence spectra of Cu_{13} nanoclusters at different temperatures.

2. Tables

Table S1. Crystal data and structure refinement for $Cu_{13}H_{10}(SR)_3(PPh_3)_7$ nanoclusters.

Compound	$Cu_{13}H_{10}(C_6H_3SFCl)_3(C_{18}H_{15}P)_7$, $3(C_6\ H_{14})$
Formula	C162 H166 Cl3 Cu13 F3 P7 S3
Formula weight	3415.30
Radiation type, wavelength (Å)	Cu Kα radiation, 1.54178 Å
Crystal system	trigonal
Space group	*R -3*
a, b, c (Å)	28.5683(3), 28.5683(3), 32.4803(6)
α, β, γ (°)	90, 90, 120
V ($Å^3$)	22957.2(6)
Z	6
Temperature (K)	173
Density (mg /m^3)	1.482
Absorption coefficient	3.870
F(000)	10470
Cell measurement theta range	4.49 to 68.25
Index ranges	-34 <=h<=34, -34 <=k<=34, -39<=l<=39
Theta range for absorption correction	0.5913 to 0.7531
Reflections collected	9362
Independent reflections	8768
Data / restraints / parameters	9362 / 276 / 644
Goodness-of-fit on F2	1.040
Final R indices [I>2sigma(I)]	R1= 0.0465, wR2= 0.1315
R indices (all data)	R1= 0.0487, wR2= 0.1335

Table S2. Selected bond lengths (Å) of $Cu_{13}H_{10}(SR)_3(PPh_3)_7$ nanoclusters.

**Atom 1**	**Atom 2**	**X-ray lengths (Å)**	**Optimized lengths (Å)**
Cu1	Cu2	2.6017(7)	2.57
Cu1	S1	2.262(1)	2.34
Cu1	Cu1	2.565(1)	2.61
Cu1	Cu3	2.5013(9)	2.51
Cu1	Cu4	2.790(1)	2.71
Cu2	Cu3	2.4904(9)	2.46
Cu2	S1	2.2850(9)	2.32
Cu2	P1	2.2364(8)	2.28
Cu2	Cu4	2.7920(8)	2.73
Cu3	Cu4	2.415(1)	2.37
Cu3	Cu5	2.5412	2.54
Cu3	Cu4	2.4704(9)	2.46
Cu4	P2	2.2402(9)	2.27
Cu5	P3	2.229	2.28
Cu1	H_t	1.65	1.67
Cu1	H_{w2}	1.62(5)	1.67
Cu2	H_{w1}	1.66(4)	1.72
Cu3	H_{w1}	1.71(5)	1.66
Cu3	H_{w2}	1.65(6)	1.69
Cu3	Hb	1.70(6)	1.70
Cu4	H_{w1}	1.74(3)	1.69
Cu4	H_{w2}	2.03(4)	1.90
Cu4	Hb	1.76(5)	1.79
Cu5	Hb	1.69	1.73
Max deviation	-	-	0.13
Average deviation	-	-	0.04

Note：H_t: the H atom at the top; H_b: the H atoms at the bottom; H_{w1}: the H atoms at the position 1 in the waist; H_{w2}: the H atoms at the position 2 in the waist.

Table S3. Crystal data analysis of $Cu_{13}H_{10}(SR)_3(PPh_3)_7$ nanoclusters.

	**Ranges**	**Averages**
Cu-Cu lengths (Å)	2.415-2.792	2.57
Cu-P lengths (Å)	2.229-2.240	2.237
Cu-S lengths (Å)	2.262-2.285	2.274
Cu-S-Cu angels (°)	69.8-69.8	69.8

Table S4. Comparison of Cu-Cu lengths in the kernel for $Cu_{13}H_{10}(SR)_3(PPh_3)_7$ nanoclusters and other previously reported Cu nanoclusters.

	Average Cu-Cu lengths (Å)	References
$Cu_{13}H_9(C_6H_3SFCl)_3(C_{18}H_{15}P)_7$	2.57	-
Bulk Cu	2.56	-
Van der Waals radii *2	2.80	-
$[Cu_{11}(TBBT)_9(PPh_3)_6]^{2+}$	3.01	1
$[Cu_{13}(S_2CN^nBu_2)_6(acetylide)_4]^+$	2.64	2
$Cu_{23}(^tBuC{\equiv}C)_{13}(CF_3COO)_6$	2.64	3
$[Cu_{25}H_{10}(SPhCl_2)_{18}]^{3-}$	2.60	4
$[Cu_{25}H_{22}(PPh_3)_{12}]^+$	2.64	5
$[Cu_{29}Cl_4H_{22}(Ph_2phen)_{12}]Cl$	2.65	6
$Cu_{30}H_{18}(S_2P(O\textit{n}Pr)_2)_{12}$	2.66	7
$[Cu_{32}(PET)_{24}H_8Cl_2](PPh_4)_2$	2.66	8

Table S5. The experimental and calculated ^{1}H NMR chemical shifts (δ) for 10 hydrides of $Cu_{13}H_{10}(SR)_3(PPh_3)_7$ nanoclusters.

**Hydrides**	**δ_{exp} (ppm)**	**δ_{cal} (ppm)**
H_{top}	0.6	1.9
H_{bottom}	2.6	2.9
$H_{waist-1}$	2.75	3.1
$H_{waist-2}$	2.78	3.4

Table S6. The attribution and transition dipole moment of the different absorption peaks in the calculated UV-vis absorption spectrum of $Cu_{13}H_{10}(SR)_3(PPh_3)_7$ nanoclusters.

Wavelength (nm)	Attribution[a]	Transition dipole moment[b]
364.1	Cu(3d)→ Cu(4p)	0.002
356.0	Cu(3d)→ Cu(4p)	0.015
342.4	Cu(3d)→ ligand π*	0.045
340.5	Cu(3d)→ ligand π*	0.025
328.2	Cu(3d)→ Cu(4p)	0.001
326.3	Cu(3d)→ ligand π*	0.031
311.8	Cu(3d)→ Cu(4p)	0.016
304.4	Cu(3d)→ ligand π*	0.025
300.9	Cu(3d)→ ligand π*	0.025

[a] Attribution：according to the maximum contribution to the absorption.

[b] Transition dipole moment: corresponding to the absorption intensity in the UV absorption spectrum.

Table S7. Cartesian coordinates of the optimized structure of $Cu_{13}H_{10}(SR)_3(PPh_3)_7$.

Atom	Cartesian coordinates			Atom	Cartesian coordinates		
Cu	-1.140727	0.980773	1.147265	H	-0.903860	4.502955	7.020957
Cu	0.071319	3.220855	0.809310	C	0.450668	2.914898	6.495071
Cu	0.943413	1.751237	-0.970113	C	0.889056	2.026731	5.523060
Cu	2.667505	0.147788	-1.228286	H	1.702447	1.331443	5.735284
Cu	0.002291	0.003261	-2.559161	C	0.281717	2.053291	4.276449
Cl	-2.460602	4.962380	4.706847	H	0.637879	1.365705	3.508735
S	-1.593498	2.910974	2.395866	C	2.417023	4.561209	2.987016
P	1.608245	4.809262	1.369205	C	3.228684	3.427205	3.118452
P	4.798460	0.478703	-1.939958	H	3.353214	2.734669	2.282421
P	0.003185	0.004850	-4.843256	C	3.859395	3.146231	4.325379
F	1.043716	2.928660	7.691503	H	4.479670	2.250450	4.406855
C	-0.767368	2.932898	3.966825	C	3.663721	3.986508	5.422077
C	-1.175846	3.808883	4.984908	H	4.138789	3.758314	6.378527
C	-0.578510	3.809345	6.245989	C	2.832191	5.098571	5.305919
H	2.657237	5.742819	6.170272	C	5.624130	-1.034104	-2.567941
C	2.209869	5.391260	4.091349	C	6.394817	-1.066376	-3.733870
H	1.558456	6.264454	4.008960	H	6.529467	-0.159642	-4.327103
C	0.829999	6.466479	1.445651	C	6.980892	-2.260573	-4.155419
C	-0.529748	6.524848	1.781251	H	7.578375	-2.274817	-5.069981
H	-1.081645	5.606628	2.003479	C	6.805261	-3.430221	-3.417489
C	-1.190522	7.750856	1.822159	H	7.264752	-4.363241	-3.751217
H	-2.249228	7.775520	2.087565	C	6.043871	-3.404546	-2.247893
C	-0.506259	8.925292	1.514660	H	5.903407	-4.309030	-1.651866
H	-1.027171	9.885260	1.535787	C	5.454847	-2.214618	-1.831775
C	0.846427	8.873673	1.176383	H	4.855334	-2.202230	-0.920105
H	1.386075	9.791810	0.933637	C	-1.307693	-1.045951	-5.568106
C	1.514049	7.650856	1.144795	C	-1.165406	-1.771786	-6.755135
H	2.571225	7.618757	0.871407	H	-0.241810	-1.694145	-7.334837
C	3.016847	5.015826	0.215492	C	-2.189045	-2.615537	-7.187507
C	4.264511	5.498881	0.628453	H	-2.065497	-3.189624	-8.108468
H	4.427097	5.757179	1.677980	C	-3.359711	-2.736876	-6.440318
C	5.305238	5.632919	-0.288412	H	-4.154099	-3.409763	-6.770805
H	6.278231	5.999141	0.045913	C	-3.510486	-2.014611	-5.256198
C	5.109573	5.284420	-1.623754	H	-4.414978	-2.126670	-4.653476
C	3.871748	4.800258	-2.044198	C	-2.486666	-1.179991	-4.818137
H	3.722338	4.507280	-3.085522	H	-2.582392	-0.651723	-3.866078
C	2.830920	4.664131	-1.128284	Cu	-0.281312	-1.479237	1.145610
H	1.866850	4.268325	-1.458463	Cu	-2.826269	-1.548199	0.801833
C	5.906434	1.041067	-0.595440	Cu	-1.985505	-0.057414	-0.974856
C	5.438385	2.064882	0.238931	Cu	-1.458837	2.238025	-1.229004
H	4.460897	2.518423	0.050576	Cl	-3.081187	-4.616617	4.692795

C	6.199449	2.490281	1.323304	S	-1.729263	-2.837790	2.389298
H	5.817256	3.281036	1.971398	P	-4.971654	-1.013315	1.357998
C	7.427047	1.888530	1.595739	P	-2.809857	3.918650	-1.941332
C	7.898908	0.870001	0.769950	F	-3.072503	-0.571143	7.686092
C	7.143770	0.446522	-0.323321	C	-2.164673	-2.135348	3.960221
H	7.516513	-0.361535	-0.956264	C	-2.722931	-2.928290	4.975297
C	5.006827	1.685503	-3.297446	C	-3.024174	-2.413380	6.236730
C	6.155962	2.464274	-3.467540	H	-3.465073	-3.042840	7.009324
H	6.979957	2.380700	-2.754171	C	-2.762276	-1.075838	6.489257
C	6.243186	3.356296	-4.535567	C	-2.208503	-0.251065	5.520294
H	7.141663	3.964841	-4.662403	H	-2.011318	0.800085	5.735308
C	5.184818	3.476987	-5.437702	C	-1.925810	-0.788165	4.273182
H	5.254775	4.181304	-6.269754	H	-1.504727	-0.135230	3.508058
C	4.036461	2.702375	-5.272352	C	-5.165115	-0.190422	2.976064
H	3.197315	2.795256	-5.965648	C	-4.588304	1.078868	3.110838
C	3.947122	1.813701	-4.203386	H	-4.047976	1.533680	2.277051
H	3.037984	1.225984	-4.057028	C	-4.663584	1.764004	4.318463
H	-4.197364	2.748575	4.402614	H	-6.243448	2.467444	-6.273727
C	-5.297249	1.173436	5.412477	C	-4.353288	2.151061	-5.276937
H	-5.339696	1.697798	6.369449	H	-4.014086	1.378925	-5.971314
C	-5.844984	-0.102255	5.293084	C	-3.539018	2.516794	-4.207480
H	-6.318327	-0.576731	6.155388	H	-2.575176	2.023596	-4.061932
C	-5.783760	-0.786061	4.077866	C	-1.911897	5.390317	-2.567623
H	-6.214673	-1.786401	3.992738	C	-2.267967	6.073574	-3.734093
C	-6.016561	-2.516996	1.431337	H	-3.119538	5.736294	-4.328494
C	-5.386698	-3.723066	1.768234	C	-1.526829	7.178570	-4.154689
H	-4.316026	-3.740763	1.992905	H	-1.812246	7.702919	-5.069685
C	-6.116650	-4.909266	1.806771	C	-0.427246	7.611854	-3.415317
H	-5.608032	-5.837930	2.072915	H	0.151095	8.476528	-3.748303
C	-7.475082	-4.905500	1.495963	C	-0.070105	6.939925	-2.245145
C	-8.107427	-3.708822	1.156797	H	0.782405	7.271010	-1.647908
C	-7.383643	-2.518331	1.127418	C	-0.806212	5.834575	-1.829899
H	-7.885091	-1.587377	0.853195	H	-0.518563	5.309643	-0.917537
C	-5.853957	0.103522	0.203649	C	1.569605	-0.604771	-5.566343
C	-6.898427	0.940156	0.615716	C	2.128763	-0.117843	-6.752219
H	-7.205300	0.950007	1.664709	H	1.600396	0.643340	-7.332321
C	-7.534779	1.774375	-0.301281	C	3.372205	-0.581671	-7.182855
H	-8.340404	2.431881	0.032282	H	3.808940	-0.186803	-8.102822
C	-7.132374	1.781757	-1.635799	C	4.061831	-1.535004	-6.435173
H	-7.619792	2.444875	-2.352996	H	5.042404	-1.885934	-6.764341
C	-6.091755	0.954434	-2.055271	C	3.509966	-2.027648	-5.252253
H	-5.760903	0.974377	-3.095761	H	4.058207	-2.755489	-4.649289
C	-5.453918	0.120778	-1.139330	C	2.274368	-1.559036	-4.815897
H	-4.627760	-0.514815	-1.468865	H	1.863500	-1.906678	-3.864618

C	-3.852139	4.595507	-0.597083	Cu	1.419250	0.495246	1.148937
C	-4.509806	3.676793	0.231755	Cu	2.752270	-1.673891	0.806099
H	-4.416668	2.604062	0.038933	Cu	1.044955	-1.689957	-0.974285
C	-5.260183	4.120932	1.316075	Cu	-1.205287	-2.381197	-1.232478
H	-5.758071	3.393491	1.959736	Cl	5.526022	-0.361806	4.706638
C	-5.348955	5.484050	1.594297	S	3.315960	-0.081079	2.397263
H	-5.921638	5.828820	2.458015	P	3.361359	-3.799804	1.361494
C	-4.697808	6.403290	0.774064	P	-1.983653	-4.390806	-1.947657
H	-4.765929	7.473024	0.985677	F	2.012532	-2.383965	7.688456
C	-3.952257	5.963431	-0.319363	C	2.921599	-0.811112	3.966497
H	-3.435283	6.691571	-0.947867	C	3.884724	-0.898081	4.984058
C	-3.958356	3.497066	-3.299924	C	3.586496	-1.417675	6.244296
C	-5.207581	4.102623	-3.469184	H	4.350055	-1.484677	7.018887
H	-5.547513	4.856793	-2.754659	C	2.297022	-1.861136	6.493012
C	-6.023573	3.733447	-4.537694	C	1.308412	-1.794177	5.521442
H	-7.000091	4.206959	-4.663685	H	0.299545	-2.151023	5.733248
C	-5.598555	2.758049	-5.441296	C	1.634983	-1.279257	4.275695
H	0.861052	-1.241367	3.508434	H	-1.426246	-7.230335	-2.762743
C	2.740229	-4.380162	2.977154	C	-0.211290	-7.079155	-4.542906
C	1.352202	-4.516404	3.106689	H	-0.133315	-8.161469	-4.670191
H	0.691233	-4.276666	2.270246	C	0.423069	-6.222460	-5.444137
C	0.792085	-4.924302	4.312216	H	0.998662	-6.634815	-6.275956
H	-0.293984	-5.013056	4.392406	C	0.326411	-4.840719	-5.278113
C	1.616419	-5.177605	5.409222	H	0.827258	-4.160194	-5.970518
H	1.180157	-5.476722	6.364610	C	-0.399322	-4.319527	-4.209374
C	2.995394	-5.012999	5.295081	H	-0.453556	-3.238414	-4.062099
H	3.639761	-5.185228	6.159889	C	-3.705949	-4.348202	-2.577318
C	3.561345	-4.617240	4.082156	C	-4.118346	-4.998977	-3.743874
H	4.643300	-4.489028	4.001323	H	-3.400191	-5.569514	-4.336248
C	5.185825	-3.952234	1.440298	C	-5.444999	-4.908514	-4.166951
C	5.913748	-2.803155	1.778831	H	-5.755388	-5.418612	-5.081872
H	5.392390	-1.867581	2.002385	C	-6.370458	-4.170837	-3.430180
C	7.305929	-2.841116	1.820885	H	-7.407759	-4.101446	-3.765182
H	7.854557	-1.935811	2.088039	C	-5.968432	-3.524556	-2.260134
C	7.983452	-4.019102	1.512130	H	-6.681665	-2.949923	-1.665021
C	7.264941	-5.165643	1.171376	C	-4.644005	-3.610621	-1.842298
H	7.792302	-6.090654	0.927735	H	-4.334260	-3.097919	-0.930191
C	5.872113	-5.135096	1.138301	C	-0.250962	1.666638	-5.565654
H	5.317970	-6.035272	0.862777	C	-0.950148	1.908352	-6.752581
C	2.838619	-5.121146	0.204331	H	-1.344324	1.070656	-7.333981
C	2.635508	-6.444510	0.614342	C	-1.169203	3.217500	-7.182575
H	2.777650	-6.716387	1.663396	H	-1.727838	3.399119	-8.103424
C	2.233330	-7.411641	-0.304695	C	-0.689667	4.290752	-6.433116
H	2.065868	-8.438450	0.027302	H	-0.875396	5.315710	-6.761762

C	2.029094	-7.065397	-1.639354	C	0.010788	4.058148	-5.249139
H	1.700492	-7.818349	-2.358249	H	0.365525	4.896399	-4.644649
C	2.226458	-5.750081	-2.056864	C	0.221884	2.753420	-4.813519
H	2.046975	-5.472397	-3.097585	H	0.726873	2.570554	-3.861601
C	2.626679	-4.781748	-1.138719	H	-8.044841	-5.837344	1.515283
H	2.763949	-3.747904	-1.466540	H	-9.171811	-3.701726	0.911541
C	-2.051631	-5.632543	-0.604389	H	-2.093656	-8.041241	2.450572
C	-0.927702	-5.746160	0.224741	H	-4.094208	-7.856552	0.977270
H	-0.043177	-5.131912	0.032159	H	9.075298	-4.045892	1.534171
C	-0.940418	-6.617987	1.309098	H	8.013043	2.210294	2.459411
H	-0.062202	-6.687811	1.953482	H	8.861486	0.396424	0.977085
C	-2.078930	-7.373116	1.586718	H	5.927605	5.374888	-2.340896
C	-3.199910	-7.265479	0.766008	H	0.033921	-1.717106	-2.344111
C	-3.188625	-6.399798	-0.327306	H	-1.504118	0.834379	-2.342809
H	-4.077227	-6.312684	-0.956073	H	1.474959	0.891784	-2.340411
C	-1.041120	-5.173679	-3.304536	H	-2.656068	-1.578101	-0.906308
C	-0.941182	-6.558162	-3.475335	H	-0.038698	3.092096	-0.899337
H	2.697218	-1.510087	-0.902154	H	-2.392514	1.212219	0.061054
H	2.245785	1.466587	0.065627	H	-0.001670	-0.001858	1.876952
H	0.147358	-2.678057	0.059490				

References

(1) Li, H.; Zhai, H.; Zhou, C.; Song, Y.; Ke, F.; Xu, W. W.; Zhu, M., Atomically Precise Copper Cluster with Intensely near-Infrared Luminescence and Its Mechanism. *J. Phys. Chem. Lett.* **2020,** *11*, 4891-4896.

(2) Chakrahari, K. K.; Liao, J. H.; Kahlal, S.; Liu, Y. C.; Chiang, M. H.; Saillard, J. Y.; Liu, C. W., $[Cu_{13}\{S_2CN^nBu_2\}_6(acetylide)_4]^+$: A Two-Electron Superatom. *Angew. Chem. Int. Ed.* **2016,** *55*, 14704-14708.

(3) Han, B.-L.; Liu, Z.; Feng, L.; Wang, Z.; Gupta, R. K.; Aikens, C. M.; Tung, C.-H.; Sun, D., Polymorphism in Atomically Precise Cu_{23} Nanocluster Incorporating Tetrahedral $[Cu_4]^0$ Kernel. *J. Am. Chem. Soc.* **2020,** *142*, 5834-5841.

(4) Sun, C. F.; Mammen, N.; Kaappa, S.; Yuan, P.; Deng, G. C.; Zhao, C. W.; Yan, J. Z.; Malola, S.; Honkala, K.; Hakkinen, H.; Teo, B. K.; Zheng, N. F., Atomically Precise, Thiolated Copper-Hydride Nanoclusters as Single-Site Hydrogenation Catalysts for Ketones in Mild Conditions. *ACS Nano* **2019,** *13*, 5975-5986.

(5) Nguyen, T. A.; Jones, Z. R.; Goldsmith, B. R.; Buratto, W. R.; Wu, G.; Scott, S. L.; Hayton, T. W., A Cu_{25} Nanocluster with Partial Cu(0) Character. *J. Am. Chem. Soc.* **2015,** *137*, 13319-24.

(6) Nguyen, T.-A. D.; Jones, Z. R.; Leto, D. F.; Wu, G.; Scott, S. L.; Hayton, T. W., Ligand-Exchange-Induced Growth of an Atomically Precise Cu_{29} Nanocluster from a Smaller Cluster. *Chem. Mater.* **2016,** *28*, 8385-8390.

(7) Barik, S. K.; Huo, S. C.; Wu, C. Y.; Chiu, T. H.; Liao, J. H.; Wang, X.; Kahlal, S.; Saillard, J. Y.; Liu, C. W., Polyhydrido Copper Nanoclusters with a Hollow Icosahedral Core: $[Cu_{30}H_{18}\{E_2P(OR)_2\}_{12}]$ (E=S or Se; R=*n*Pr, *i*Pr or *i*Bu). *Chem. Eur. J.* **2020,** *26*, 10471-10479.

(8) Lee, S.; Bootharaju, M. S.; Deng, G.; Malola, S.; Baek, W.; Hakkinen, H.; Zheng, N.; Hyeon, T., $[Cu_{32}(PET)_{24}H_8Cl_2](PPh_4)_2$: A Copper Hydride Nanocluster with a Bisquare Antiprismatic Core. *J. Am. Chem. Soc.* **2020,** *142*, 13974−13981.
